# Parallel Homochiral and Anti‐Parallel Heterochiral Hydrogen‐Bonding Interfaces in Multi‐Helical Abiotic Foldamers

**DOI:** 10.1002/anie.201912805

**Published:** 2019-12-12

**Authors:** Daniela Mazzier, Soumen De, Barbara Wicher, Victor Maurizot, Ivan Huc

**Affiliations:** ^1^ Department of Pharmacy and Centre for Integrated Protein Science Ludwig-Maximilians-Universität Butenandtstrasse 5–13 81377 Munich Germany; ^2^ CBMN Laboratory Université de Bordeaux CNRS, IPB Institut Européen de Chimie et Biologie 2 rue Escarpit 33600 Pessac France; ^3^ Department of Chemical Technology of Drugs Poznan University of Medical Sciences Grunwaldzka 6 60–780 Poznan Poland

**Keywords:** foldamers, helix bundles, molecular design, structure elucidation, tertiary structures

## Abstract

A hydrogen‐bonding interface between helical aromatic oligoamide foldamers has been designed to promote the folding of a helix‐turn‐helix motif with a head‐to‐tail arrangement of two helices of opposite handedness. This design complements an earlier helix‐turn‐helix motif with a head‐to‐head arrangement of two helices of identical handedness interface. The two motifs were shown to have comparable stability and were combined in a unimolecular tetra‐helix fold constituting the largest abiotic tertiary structure to date.

## Graphical abstract

**Turn right, then left, left again, and then right**: Both *P*‐ and *M*‐helices can be assembled in the same abiotic tertiary foldamer structures without any control of absolute configurations thanks to the design of *P*/*P*, *M*/*M*, and *P*/*M* helix/helix hydrogen‐bonding interfaces.

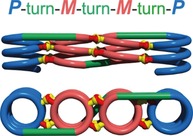

Foldamer research has shown that secondary structures, such as isolated helices or β‐strands, occur in a great variety of synthetic backbones.^1^ In contrast, the design of tertiary folds is a considerable challenge. This challenge is worth pursuing because tertiary folding is the level at which sophisticated functions emerge in proteins and the same may be expected for foldamers. The way is being paved by impressive progress in protein design^2^ and increasing mastery in programming binding interfaces between peptidic structures, in particular within peptide helix bundles.2b, 2c, 3 For instance, helix bundles have been reported in peptidomimetics, such as β‐peptides^4^ and β‐ureas.^5^ We have recently introduced the first abiotic tertiary folds, that is, from backbones that do not relate to peptides or nucleotides.^6^ We used the stable helices formed by aromatic oligoamides of 8‐amino‐quinolinecarboxylic acid^7^ (**Q** in Figure 1) and 6‐aminomethylpyridine carboxylic acid^8^ as well‐defined modules and introduced hydroxy groups at precise positions at their periphery (**X** and **Y** in Figure 1) to promote inter‐helix hydrogen bonding with amide carbonyl groups (Figure 2 a). Various types of helix bundling were observed, including parallel trimers and dimers, and tilted dimers.^6^, ^9^ As opposed to biotic tertiary folds that form in water and are often driven by hydrophobic effects, these folds form in organic solvents. All these assemblies were homochiral, that is, they involved helices that have the same handedness. Further progress in tertiary structures design will primarily rest on the orchestration of interactions between secondary folds, a far‐from‐trivial endeavor. Herein we introduce a binding interface between helices of opposite handedness. Unlike what was recently shown in heterochiral peptide bundling,^10^ we demonstrate the equivalence of parallel homochiral and anti‐parallel heterochiral abiotic helix association. We also show how the two patterns can be combined within the same tertiary fold without having to consider the stereochemistry at each unit, as it would in a peptide.

**Figure 1:**
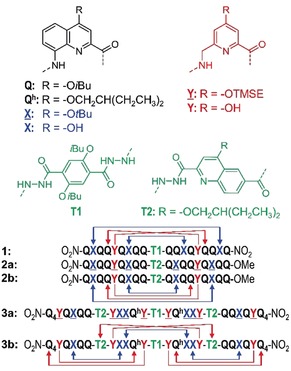
Structures of units **Q**, **Q^h^**, **X**, **Y**, **T1**, **T2**, and foldamer sequences. **X** and **Y** are the protected precursors of hydrogen‐bonding units **X** and **Y**. Sequences are labelled “**a**” when protected and “**b**” when deprotected. Sequences end with an 8‐nitro group at their N‐terminus: this group is noted in the replacement of the NH group at N‐terminal **Q** units. The **T1** unit constitutes an inversion of C→N sequence polarity; sequences that contain **T1** thus have two N termini. The arrows indicate the hydrogen‐bonding patterns between the helices and point towards the hydrogen‐bond acceptor. TMSE=2‐trimethylsilylethyl.

**Figure 2:**
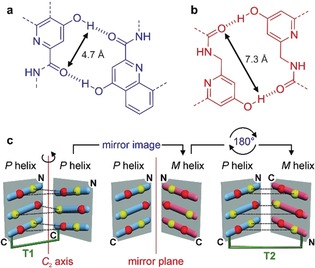
Hydrogen‐bonding patterns involving **X** (a) and **Y** (b) units. Double‐headed arrows indicate the different distances between the two hydrogen bonds for both **X** and **Y** units, as represented in (c). c) Helical net diagrams depicting hydrogen‐bond interfaces between helices. The arrays of six hydrogen‐bond donors (yellow) and acceptors (red) belonging to **X** (top and bottom) and **Y** (middle) units are approximated to belong to two planes facing each other. Hydrogen bonds are shown as dotted black lines. Blue and pink rods represent the rims of the helices and are tilted in different directions according to their *P* or *M* helix handedness. A hydrogen‐bond array between parallel homochiral helices (left) can be transformed in an equivalent array between anti‐parallel heterochiral helices (right). In the plane symmetrical central structure, hydrogen‐bond donors (reciprocally acceptors) face each other and no hydrogen bond forms: inverting either helix handedness or sequence orientation makes hydrogen bonding impossible, whereas changing both is a productive transformation. The *C*_2_ axis in the diagram at left is best seen in the crystal structure shown in Figure 4 a.

Turn unit **T1** (Figure 1) has been shown to promote homochiral parallel helix bundling between two identical helical segments attached at their C‐terminus as, for example in sequence **1** (Figure 1).^6^ Molecular modelling was used to design **1**: it allowed us to adjust the positions of hydrogen‐bond donors and led to the replacement of some **X** units by **Y** to avoid possible steric repulsions. A diagram of the helix–helix interface illustrates how the hydrogen‐bond donors and acceptors may face each other (Figure 2 c, left). This diagram also shows that hydrogen bonding occurs despite the helices having the same handedness: the slope of the main chain (i.e. the angle between its tangent and a plane perpendicular to the axis) should in principle result in some distance between hydrogen‐bond donors and acceptors placed on two helices of identical handedness. Yet the large helix diameter and the resulting moderate slope (ca. 15°) are such that hydrogen bonding takes place. An extension of this observation is that an anti‐parallel heterochiral helix dimer (Figure 2 c, right) should not only give rise to a similar hydrogen‐bonding pattern, but in fact lead to a better match between the positions of the donors and acceptors because the helical chains have their tangent parallel to each other at the interface, that is, their slopes have opposite signs (Figure 2 c).^11^ To test this prediction, we designed turn unit **T2** and sequence **2** (Figure 1). It should be noted that sequence **2** contains the very same nonameric helix segment as **1** but that one of the two is now attached to the turn at its N‐terminus.

The synthesis of the Fmoc‐protected version of **T2** is described in the Supporting Information. In anticipation of the preparation of long oligomers, we developed a solid‐phase fragment condensation (SPFC) approach (Figure 3 a). Fragments **A** and **B** were synthesized using previously reported solid‐phase synthesis (SPS) methods.8c, 12 Fragment **A** was then cleaved from the resin, purified and coupled to **T2**‐terminated fragment **B** still on the resin. To prepare **2 a**, two identical fragments were condensed. Using mild resin cleavage conditions, oligomer **4** was obtained as a free carboxylic acid with its side chains protections, and was then converted into the corresponding methyl ester **2 a**.

**Figure 3:**
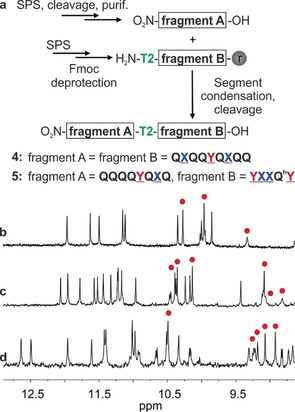
a) Scheme of the synthesis of compounds **4** and **5** via solid‐phase fragment condensation (SPFC). Extract of ^1^H NMR spectra (500 MHz, CDCl_3_) showing the NH and OH resonances of **1** ^[9]^ (b), **2 b** (c) and **3 b** (d). The red dots indicate signals corresponding to OH protons.

The ^1^H NMR spectrum of **2 a** in CDCl_3_ shows two sets of signals, suggesting the coexistence of *PM* and *PP*/*MM* conformers in solution (Figure S1a in the Supporting Information), as was previously observed for structures containing **T1**.^6^, ^9^ On the contrary, the deprotected sequence **2 b** shows one set of sharp NMR signals including for OH resonances (Figure 3 c). The spectrum is similar to that of **1** (Figure 3 b) and indicative of a well‐folded helix‐turn‐helix motif. A crystal structure of **2 b** confirmed the formation of the anti‐parallel heterochiral helix dimer (Figure 4 b). The resemblance of the hydrogen‐bonding interface in this structure with that of **1** (Figure 4 a), is striking. Despite the change of one helix handedness and orientation, the hydrogen‐bond donors and acceptors are found at very similar positions (Figures S2–S3). The stability of the hydrogen‐bonding interfaces was then assessed upon monitoring the effect adding [D_6_]DMSO into CDCl_3_ solutions. Because of the rigidity of the aromatic helices, chelate effects are observed between the hydrogen‐bonding units that results in an all‐or‐nothing behavior: the six hydrogen bonds are disrupted all at once through a small change of DMSO concentration. Remarkably, this transition occurred with identical amounts of DMSO (ca. 20 % vol/vol, Figures S4–S7) for **1** and **2 b**, showing comparable strengths of the parallel homochiral and anti‐parallel heterochiral hydrogen‐bonding interfaces.

**Figure 4:**
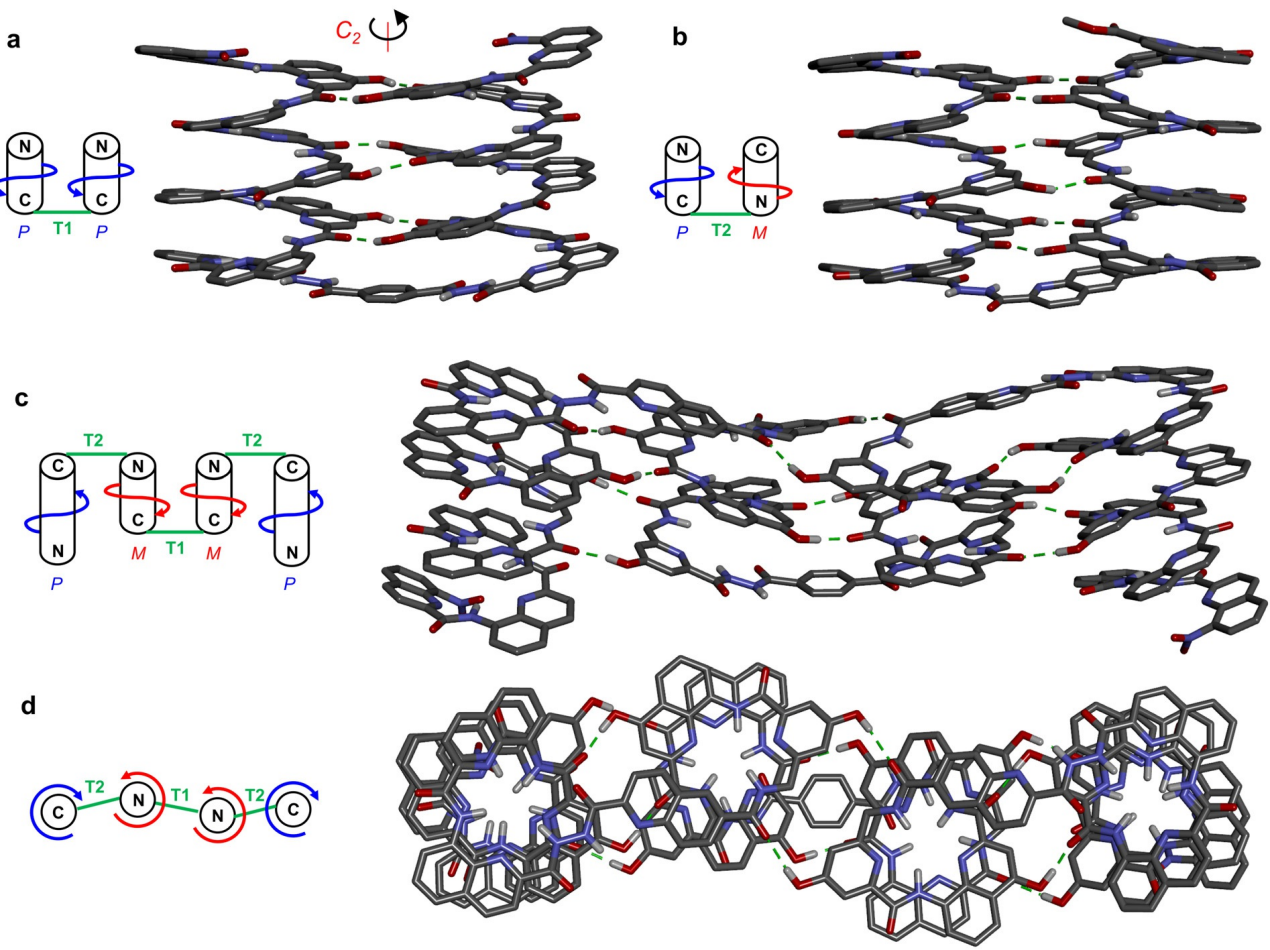
Crystal structures of compounds **1^[6]^** (a) and **2 b** (b). Front view (c) and top view (d) of energy‐minimized molecular model of **3 b**. Cartoons indicate helix handedness, C and N‐termini. Side chains of **Q**, **Q^h^**, **T1**, and **T2**, included solvent molecules and most hydrogen atoms have been omitted for clarity. For crystallographic details and the CCDC number see the Supporting Information.

Differences from peptide helical bundles should be noted: in peptides, bundling is mostly known between α‐helices of identical handedness which, to best match at their binding interfaces, generally coil around one another. Studies on heterochiral peptide helix bundling^10^ and in particular some recent work by Gellman et al.,10a, 10b show that homochiral and heterochiral peptide helix interfaces are not equivalent, notably because coil‐coiling is not conducive of better complementarity in heterochiral bundles. In contrast, the rigidity of the aromatic helices hampers coiling, at least over short distances, and strictly parallel arrangements form when mediated by turn units, such as **T1** and **T2**. However, aromatic helices may slightly change their local curvature, so as to optimize inter‐helix interactions: helix curvature in the structures of **1** and **2 b** is not rigorously constant and identical to that of relaxed helices not involved in bundling.

In compounds **1** and **2 b**, helix handedness control is relative, not absolute, and guided only by strand orientation as imposed by the turn unit, and by hydrogen‐bonding complementarity. This should in principle allow for the combination of both parallel‐homochiral and anti‐parallel‐heterochiral motifs in the same tertiary structure without having to consider the nature of stereogenic centers at each unit as it would in peptides. We challenged this possibility through the design of sequence **3 b** (Figure 1). As shown in Figure 4 c,d, **3 b** is expected to fold in a sequence of four contiguous helices having either identical or opposite handedness depending on whether they are separated by **T1** or **T2**, with the central **YXXQ^h^Y** helical segments each bearing two independent hydrogen‐bonding interfaces, one homochiral, and one heterochiral. In the design of **3 b**, we made use of helices of different length to avoid creating an extended aromatic surface that might promote aggregation and reduce solubility. Similarly, we introduced **Q^h^** units with a longer branched alkyl chain inside the sequence to promote solubility (Figure 1). Oligomer **3 a** was synthesized combining SPFC (Figure 3 a) and solution‐phase synthesis for the final coupling of **T1** with **5**. The ^1^H NMR spectrum of protected compound **3 a** was complex due to the presence of multiple turn units (Figure S1 b) and thus of different conformational stereoisomers (*PMMP*, *PMMM*, *MMMM*, *PMPM*, *PMPP*, *MMPP* and their enantiomers). After removal of the side chain protecting groups, a sharp spectrum with only one set of signals was observed for **3 b** (Figure 3 d). Even though an unambiguous structure elucidation could not be achieved in solution or in the solid state, these observations altogether suggest that **3 b** is present in solution in a well‐defined folded conformation.

In conclusion, we have introduced a new well‐defined abiotic helix–helix hydrogen‐bonding interface and showed that tertiary structures combining different interfaces can be designed, resulting in predictable helix‐turn‐helix structures composed of helices of different handedness and orientation, a pattern difficult to reach with simple peptides. We are currently expanding this work to interfaces between tilted, that is, non‐parallel, helix multimers and will report our progress in due course.

## Conflict of interest

The authors declare no conflict of interest.

## Supporting information

As a service to our authors and readers, this journal provides supporting information supplied by the authors. Such materials are peer reviewed and may be re‐organized for online delivery, but are not copy‐edited or typeset. Technical support issues arising from supporting information (other than missing files) should be addressed to the authors.

Supplementary
