# Supplementary material for: Parallel Homochiral and Anti‐Parallel Heterochiral Hydrogen‐Bonding Interfaces in Multi‐Helical Abiotic Foldamers
Source: Angew Chem Int Ed Engl. 2019 Dec 12;59(4):1606–10. doi: 10.1002/anie.201912805 (PMC7004161; doi:10.1002/anie.201912805)
Supplement: Supplementary file 1 — Supplementary [file ANIE-59-1606-s001.pdf]

Angewandte Chemie
Eine Zeitschrift der Gesellschaft Deutscher Chemiker

Supporting Information

Parallel Homochiral and Anti-Parallel Heterochiral Hydrogen-Bonding Interfaces in Multi-Helical Abiotic Foldamers

*Daniela Mazzier, Soumen De, Barbara Wicher, Victor Maurizot, and Ivan Huc**

anie_201912805_sm_miscellaneous_information.pdf

Table of Contents **page**

1. Supplementary figures 2
2. Supplementary methods 7
    1. Nuclear magnetic resonance spectroscopy 7
    2. X-ray crystallography 7
3. Synthetic schemes 10
4. Experimental procedures 13
    1. General methods 13
    2. Synthesis of small molecules 14
    3. Solid phase synthesis general methods 18
    4. Synthesis of oligomers 18
5. References 22
6. ^{1}H and ^{13}C NMR spectra of new compounds 23
7. RP-HPLC profiles of oligomers 35

1. Supplementary figures

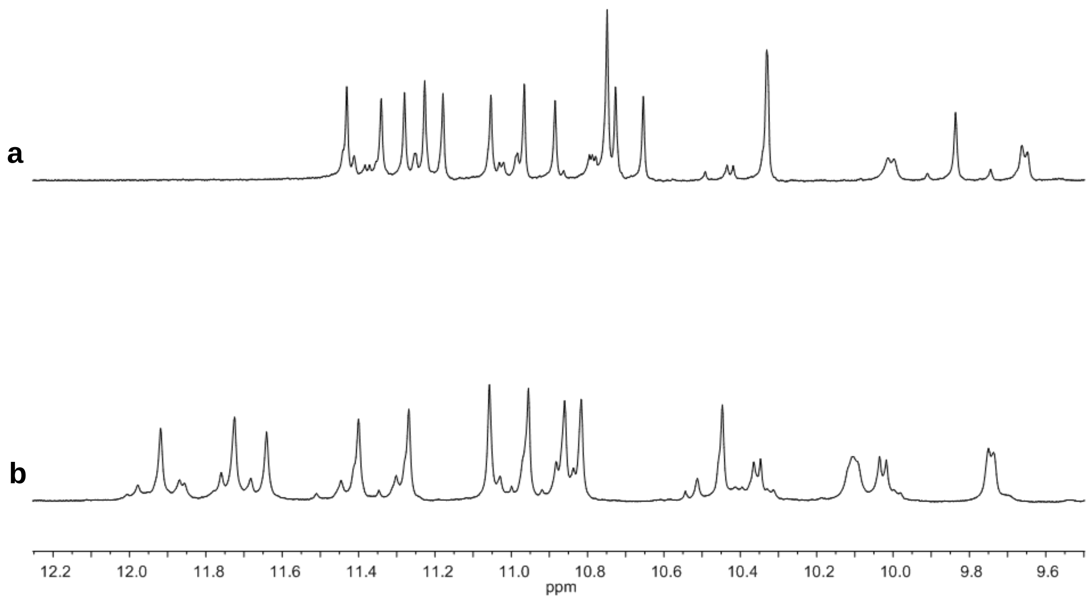

Figure S1 Extract of ^{1}H NMR spectra (500 MHz, $CDCl_3$) of **2a** (a) and **3a** (b).

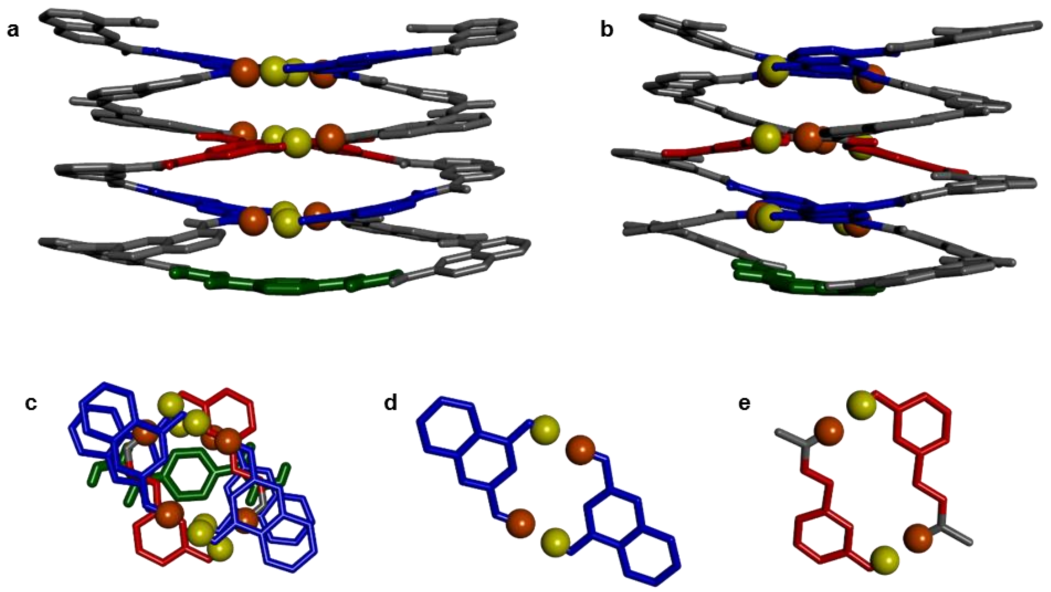

Figure S2 Crystal structure of **1**. Side views (**a**, **b**), top view of the stack of **X**, **Y** and **T1** units (**c**). Hydrogen-bonded pairs of **X** units (**d**) and **Y** units (**e**) translated horizontally from the stack. The hydroxyl protons and carbonyl oxygen atoms of the hydrogen-bonding arrays are shown as yellow and orange balls, respectively. **X**, **Y** and **T1** units are shown as blue, red and green tubes. Side chains of **Q** and **T1**, included solvent molecules and most hydrogen atoms have been omitted for clarity.

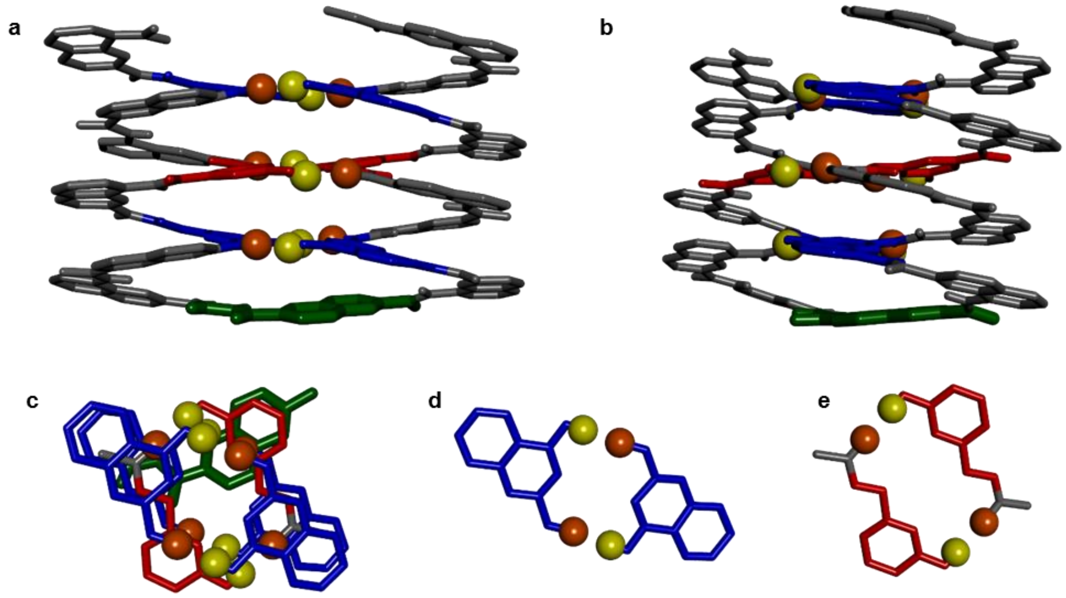

Figure S3 Crystal structure of **2b**. Side views (**a**, **b**), top view of the stack of **X**, **Y** and **T2** units (**c**). Hydrogen-bonded pairs of **X** units (**d**) and **Y** units (**e**) translated horizontally from the stack. The hydroxyl protons and carbonyl oxygen atoms of the hydrogen-bonding arrays are shown as yellow and orange balls, respectively. **X**, **Y** and **T2** units are shown as blue, red and green tubes. Side chains of **Q** and **T2**, included solvent molecules and most hydrogen atoms have been omitted for clarity.

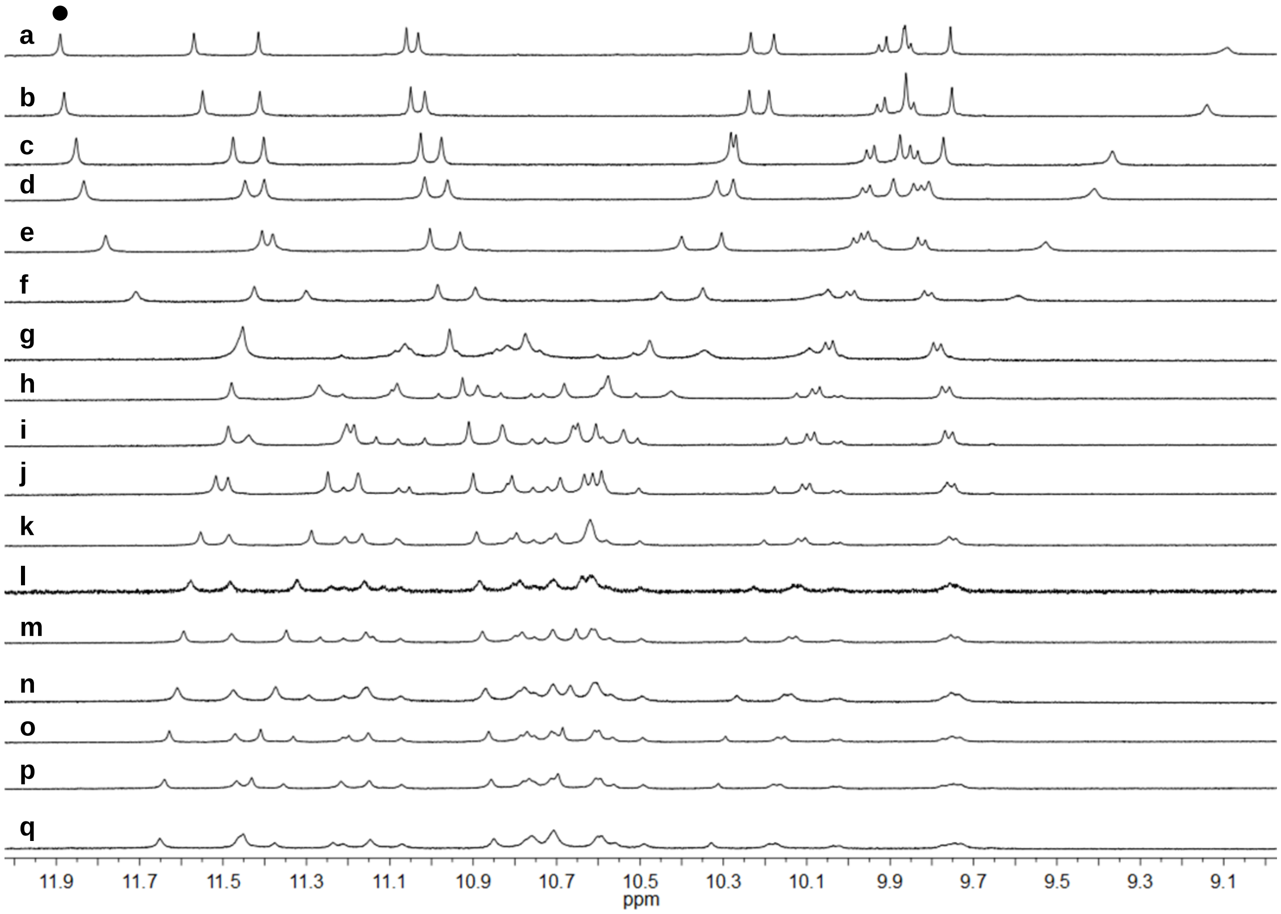

Figure S4 Part of the ^{1}H NMR spectra (500 MHz) showing amide resonances of **1** in $CDCl_3$/DMSO-d_6. The volume percentages of DMSO-d_6 are 4 (a), 8 (b), 10 (c), 12 (d), 14 (e), 16 (f), 18 (g), 20 (h), 22 (i), 24 (j), 26 (k), 28 (l), 30 (m), 32 (n), 34 (o), 36 (p) and 38% (q) respectively. The chemical shift variations of the signal marked with a dot is shown in Figure S5.

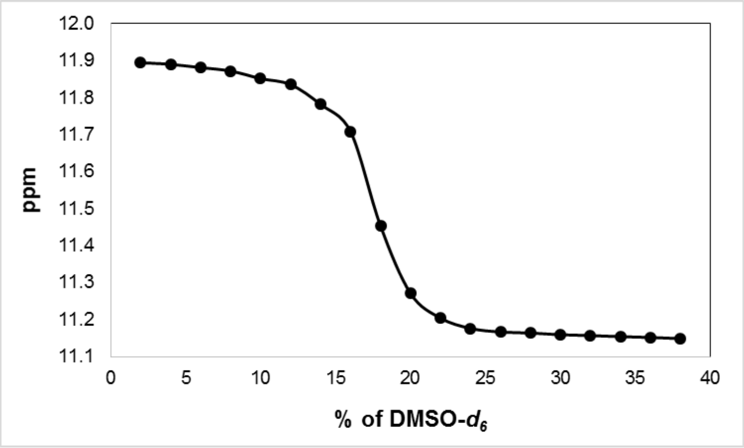

Figure S5 NMR chemical shift of amide NH proton of **1** as a function of the volume percent of DMSO-d_6 in $CDCl_3$.

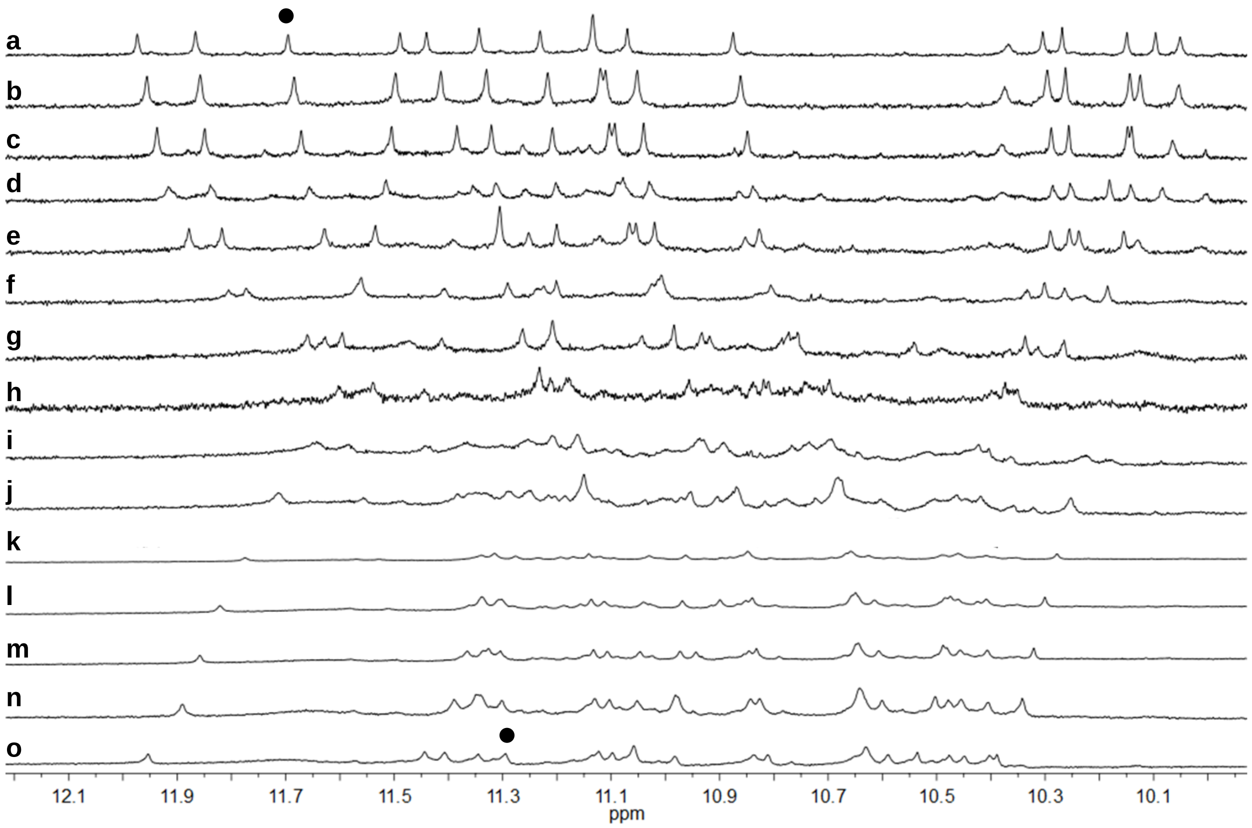

Figure S6 Part of the ^{1}H NMR spectra (500 MHz) showing amide resonances of **2b** in $CDCl_3$/DMSO-*d*$_6$. The volume percentages of DMSO-*d*$_6$ are 4 (a), 8 (b), 10 (c), 12 (d), 14 (e), 16 (f), 18 (g), 20 (h), 22 (i), 24 (j), 26 (k), 28 (l), 30% (m), 32 (n), 34 (o) and 36% (p) respectively. The chemical shift variations of the signal marked with a dot is shown in Figure S7.

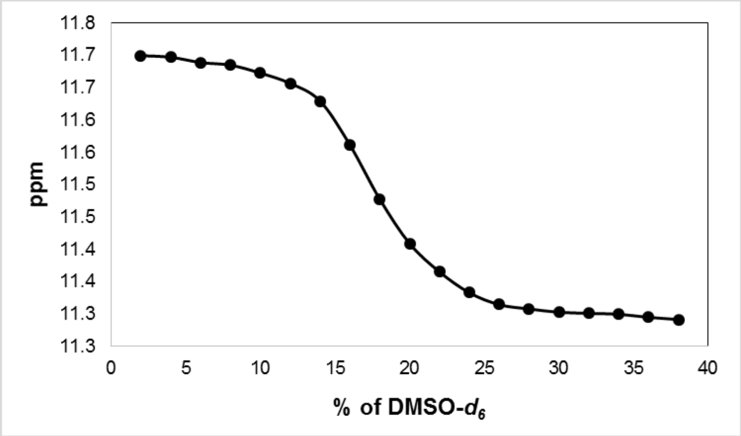

Figure S7 NMR chemical shift of amide NH protons of **2b** as a function of the volume percent of DMSO-*d*$_6$ in $CDCl_3$.

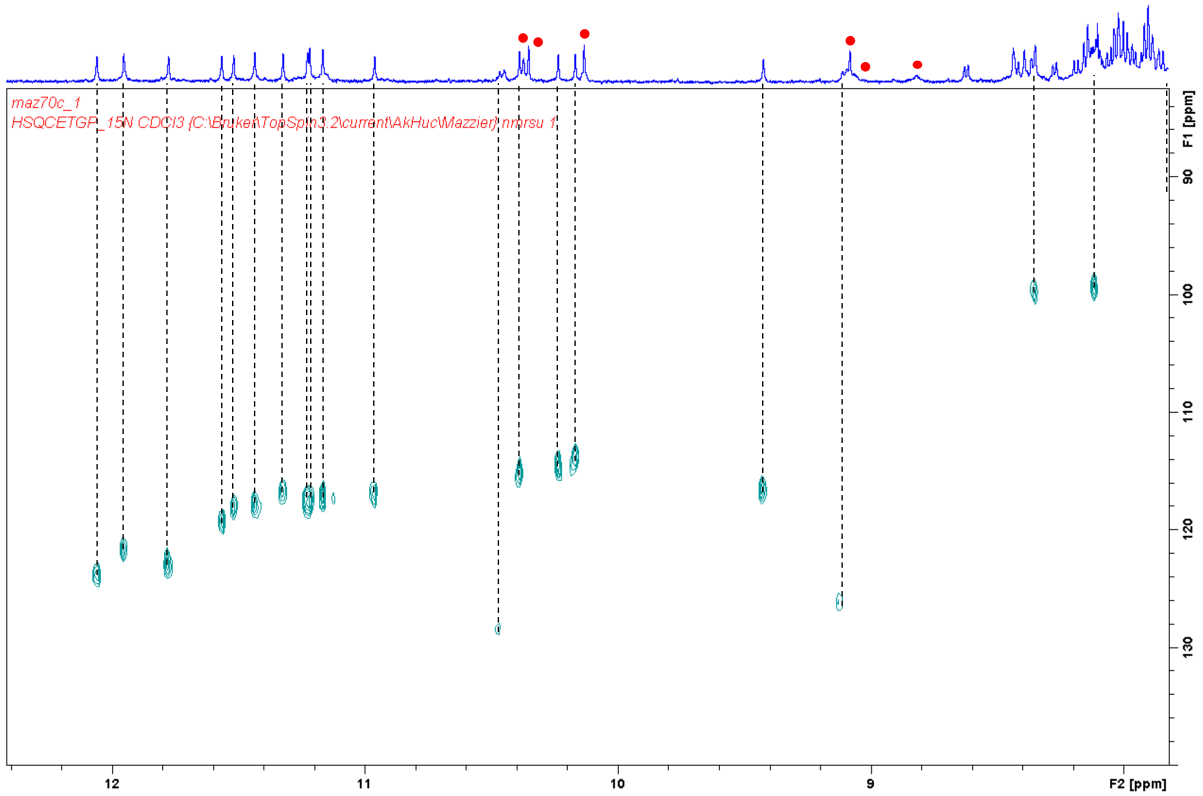

Figure S8 ^{15}N-^{1}H HSQC (500 MHz, $CDCl_3$) of **2b**. Only NH resonances correlate, red dots indicate the signals of OH protons.

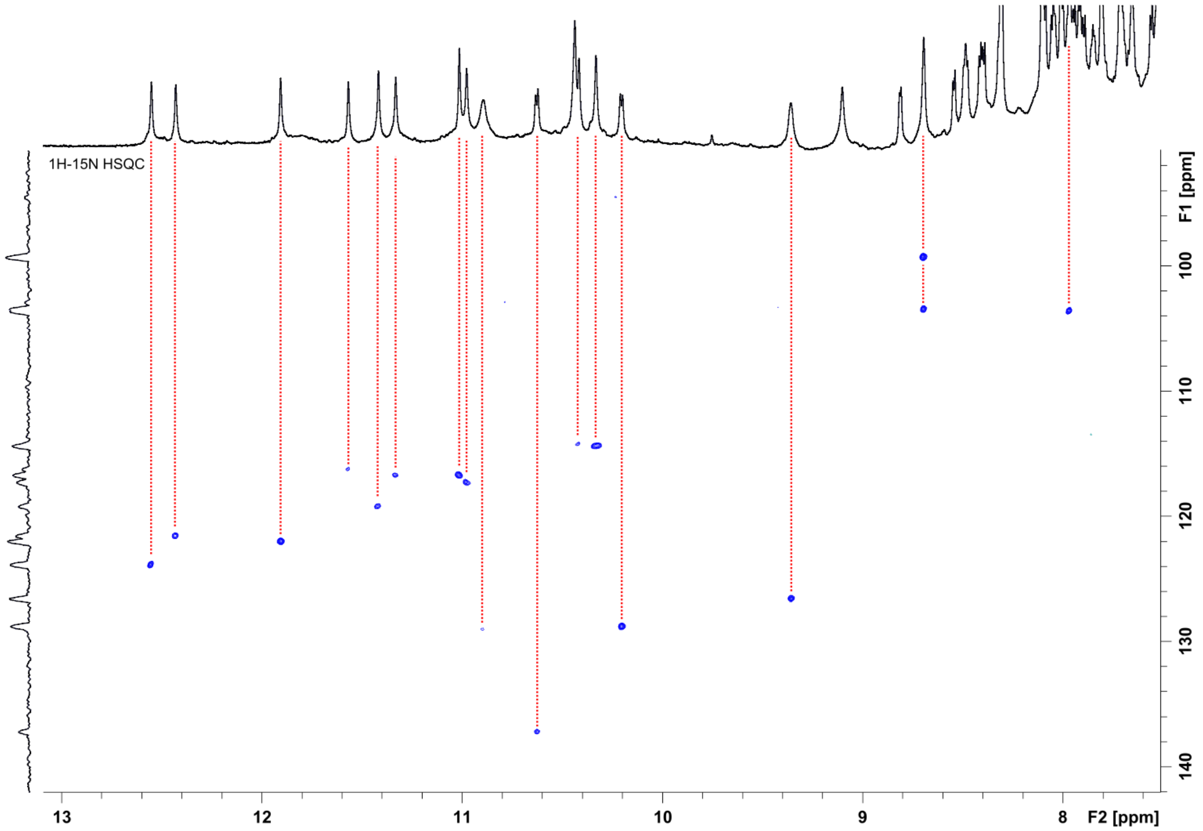

Figure S9 ^{15}N-^{1}H HSQC (700 MHz, $CDCl_3$) of **3b**. Only NH resonances correlate.

2. Supplementary methods

2.1 Nuclear magnetic resonance spectroscopy

NMR spectra were recorded on different NMR spectrometers: (I) an Avance II NMR spectrometer (Bruker BioSpin) with a vertical 7.05 T narrow-bore/ultrashield magnet operating at 300 MHz for ^{1}H observation and 75 MHz for ^{13}C observation by means of a 5-mm direct BBO H/X probe with Z gradient capabilities; (II) an Avance III HD NMR spectrometer 400 MHz (Bruker BioSpin); (III) an Avance III HD NMR spectrometer 500 MHz (Bruker BioSpin) with CryoProbe™ Prodigy.

Chemical shifts are described in part per million (ppm, δ) relative to the ^{1}H residual signal of the deuterated solvent used. ^{1}H NMR splitting patterns with observed first-order coupling are entitled as singlet (s), doublet (d), triplet (t), quartet (q) or broad singlet (bs). Coupling constants (*J*) are reported in hertz.

2.2 X-ray crystallography

Single crystal X-ray diffraction data for **2b** were collected with a Rigaku FRX rotating anode (2.9 kW) diffractometer at the IECB x-ray facility (UMS 3033 – UMS001). CuKα radiation monochromated with high flux Osmic Varimax mirrors was used for data collection. The x-ray source is equipped with a Dectris Pilatus 200K detector and partial chi goniometer. During data collection the crystal was cooled with a nitrogen gas stream to 100(2) K. Data were processed with the CrysAlis PRO[1] software. The structure was solved with the ShelXT0[2] structure solution program using Intrinsic Phasing. Using Olex2,[3] the structure was refined with the ShelXL[4] refinement package using Least Squares minimization.

Only non-H atoms of the backbones were refined with anisotropic displacement parameters. Positions of the hydrogen atoms of the O-H groups were deducted on the basis of the possible hydrogen bonding interactions. The O-H distances were restrained to 0.84 Å and displacement parameters were set equal to 1.5Ueq(O). For backbones H atoms were placed geometrically and constrained depending on their environment. Those H-atoms were refined in the riding-model approximation, with Uiso(H)=1.2Ueq(CH, CH2, NH). DFIX, AFIX, FLAT, EADP and DELU instructions were employed to model geometry of the molecules and temperature parameters. In the electron density map, the highest residual electron density peak Q1 has a value 1.56 ē and is in close proximity to the hydrazide function of the turn unit. The Q1···N20C distance (2.287 (1) Å) is too short to assign Q1 to a water molecule, as was done for O1W. Q1 might be cation (Na+ or Li+), however, deprotonation of hydrazide does not make much chemical sense so Q1 was left as is.

Refinement of large foldamer crystal structures faces problems usually observed in macromolecular crystallography, i.e. large volume fractions of disordered solvent molecules, weak diffraction intensity, incompleteness of the data, moderate or low resolution. Thus, it is not surprising that a number of A-level and B-level alerts were detected using IUCR's checkcif algorithm. These alerts are inherent to the data and refinement procedures and do not reflect errors. Rather, they illustrate the limited practicality of the checkcif tool for medium size molecule crystallography. They are listed below and have been divided into two groups.

Group 1 alerts illustrate weak quality of the data and refinement statistics if compared to that expected for small molecule structures from highly diffracting crystals:

THETM01_ALERT_3_A The value of sine(theta_max)/wavelength is less than 0.550
PLAT023_ALERT_3_A Resolution (too) Low [sin(theta)/Lambda < 0.6].
PLAT934_ALERT_3_A Number of (Iobs-Icalc)/SigmaW > 10 Outliers
PLAT084_ALERT_3_A High wR2 Value (i.e. > 0.25)
PLAT082_ALERT_2_B High R1 Value .... 0.20 Report
PLAT234_ALERT_4_B Large Hirshfeld Difference C65B --C66B . 0.29 Ang.
PLAT241_ALERT_2_B High 'MainMol' Ueq as Compared to Neighbors
PLAT242_ALERT_2_B Low 'MainMol' Ueq as Compared to Neighbors of
PLAT340_ALERT_3_B Low Bond Precision on C-C Bonds .... 0.01755 Ang.

Group 2 alerts is connected with decision made during refinement and explained below:

PLAT201_ALERT_2_A Isotropic non-H Atoms in Main Residue(s)
As mentioned above not all atoms were refined with ADP
PLAT043_ALERT_1_A Calculated and Reported Mol. Weight Differ by .. 344.10 Check
Not all H atoms were determined but they were used in SFAC calculation
PLAT602_ALERT_2_A VERY LARGE Solvent Accessible VOID(S) in Structure !
It was not possible to determined severely disordered solvent molecules
PLAT430_ALERT_2_B Short Inter D...A Contact
These contacts are connected with dummy O atoms introduced into refinement
PLAT306_ALERT_2_B Isolated Oxygen Atom (H-atoms Missing ?)
Dummy O atoms were introduced into refinement
PLAT315_ALERT_2_B Singly Bonded Carbon Detected (H-atoms Missing)
Not all H atoms were introduce into refinement

Table S1. Crystal data and refinement details for **2b**.

**Identification code**	**2b**
**Chemical formula**	$2(C_{240}H_{225}N_{39}O_{42})\cdot H_2O\cdot 12(C_3H_6O)\cdot 6.3(CHCl_3)\cdot 12.66O$*
**Formula weight**	10324.68
**Temperature**	100(2)
**Wavelength**	1.54178 Å
**Crystal system**	Monoclinic
**Space group**	*C*2/c
**Unit cell dimensions**	a=57.550 (1), α=90
	b=31.4689 (5), β=96.713 (2)
	c=28.6463 (4), γ=90
**Volume**	51524 (2)
Z	4
**Density (calculated)**	1.331
**Absorption coefficient**	1.65
**Absorption correction**	Multi-scan
**Crystal size**	0.10 × 0.06 × 0.02
**Index ranges**	*h = -57→58, k = -31→31, l = -28→27*
**Completeness to theta = 51.12º**	99.2
**Reflections collected**	112713
**Reflections observed [*I* > 2σ(*I*)]**	19506
**R_{int}**	0.032
**Data/parameters/restrains**	27547/2838/150
**Goodness-of-fit on F^2**	2.47
**Final R indices [*I* > 2σ(*I*)]**	R1 = 0.2014, wR2 = 0.5290
**R indices (all data)**	R1 = 0.2280, wR2 = 0.35543
**Lasgest diff. peak and hole**	1.59, -1.28
**CCDC #**	1955168

*Unrecognized electron density was introduced to the refinement as a dummy oxygen atoms.

Table S2. Selected hydrogen-bond parameters. Atom numbers are those of the cif file.

D—H···*A*	*D*—H (Å)	H···*A* (Å)	*D*···*A* (Å)	*D*—H···*A* (°)
O25C—H25C···O5C	0.84	1.83	2.659 (12)	169
O4C—H4C···O26C	0.84	1.86	2.631 (11)	152
O8C—H8C···O21C	0.84	1.99	2.799 (11)	163
O22C—H22C···O7C	0.84	1.98	2.798 (10)	163
O11C—H11C···O19C	0.84	2.01	2.769 (10)	150
O18C—H18C···O12C	0.84	1.85	2.687 (10)	175

3. Synthetic schemes

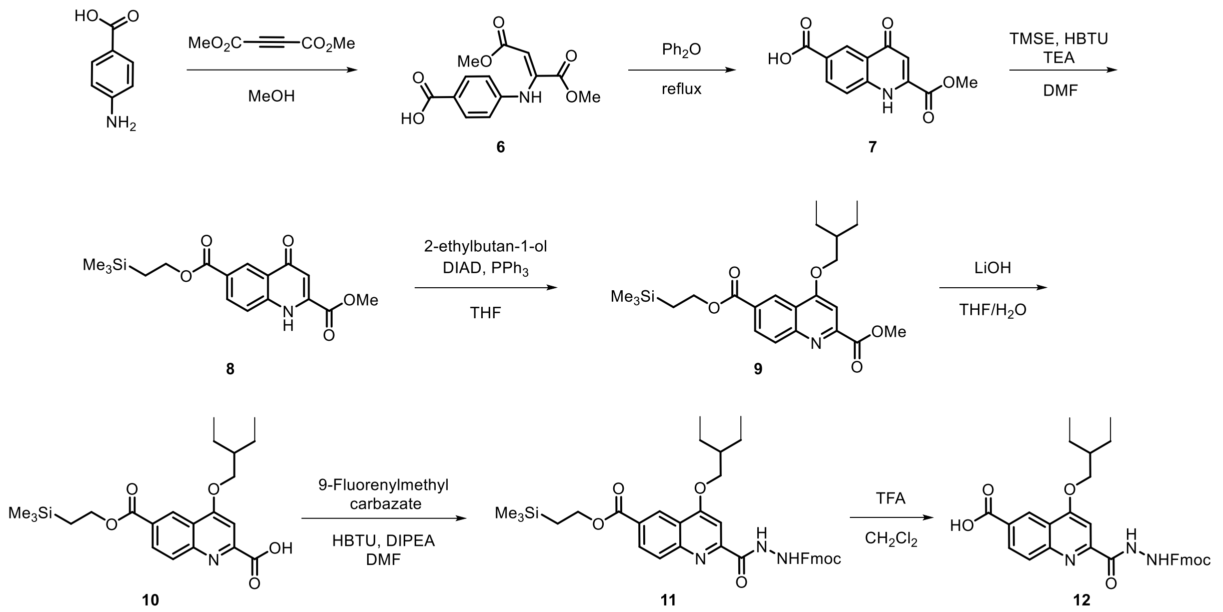

Scheme S1: Synthesis of **12**

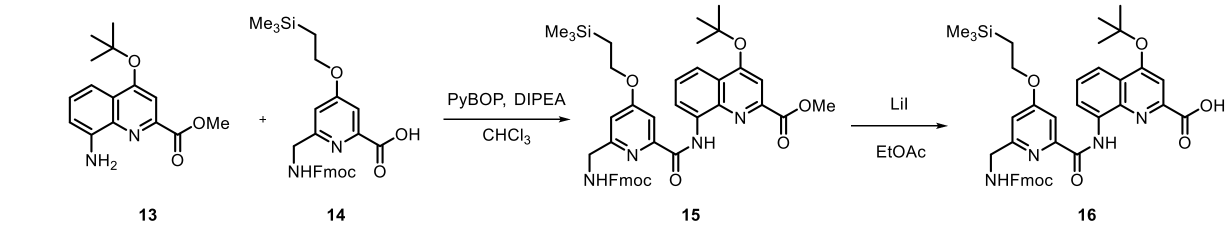

Scheme S2: Synthesis of **16**

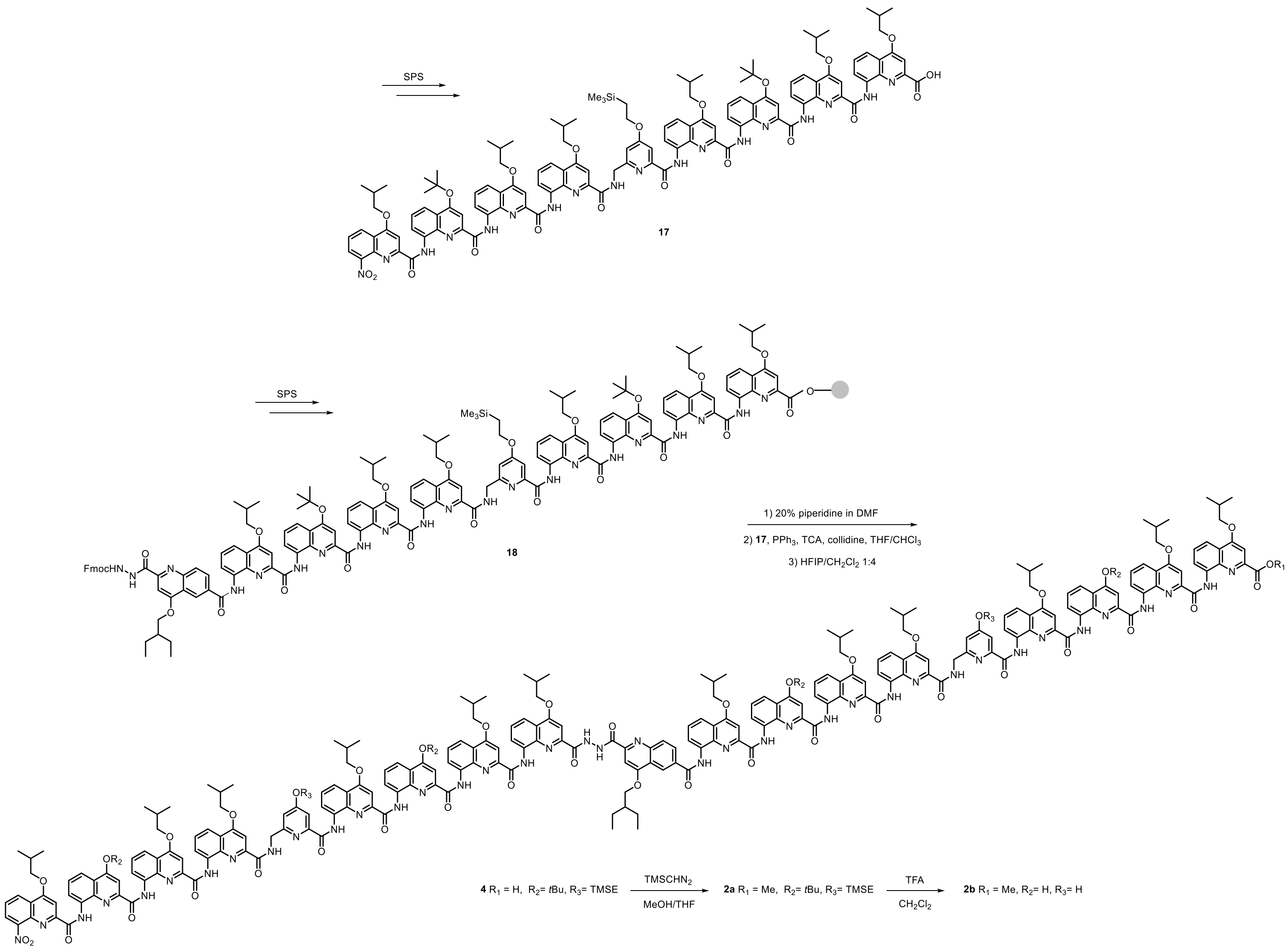

Scheme S3: Synthesis of **2b**

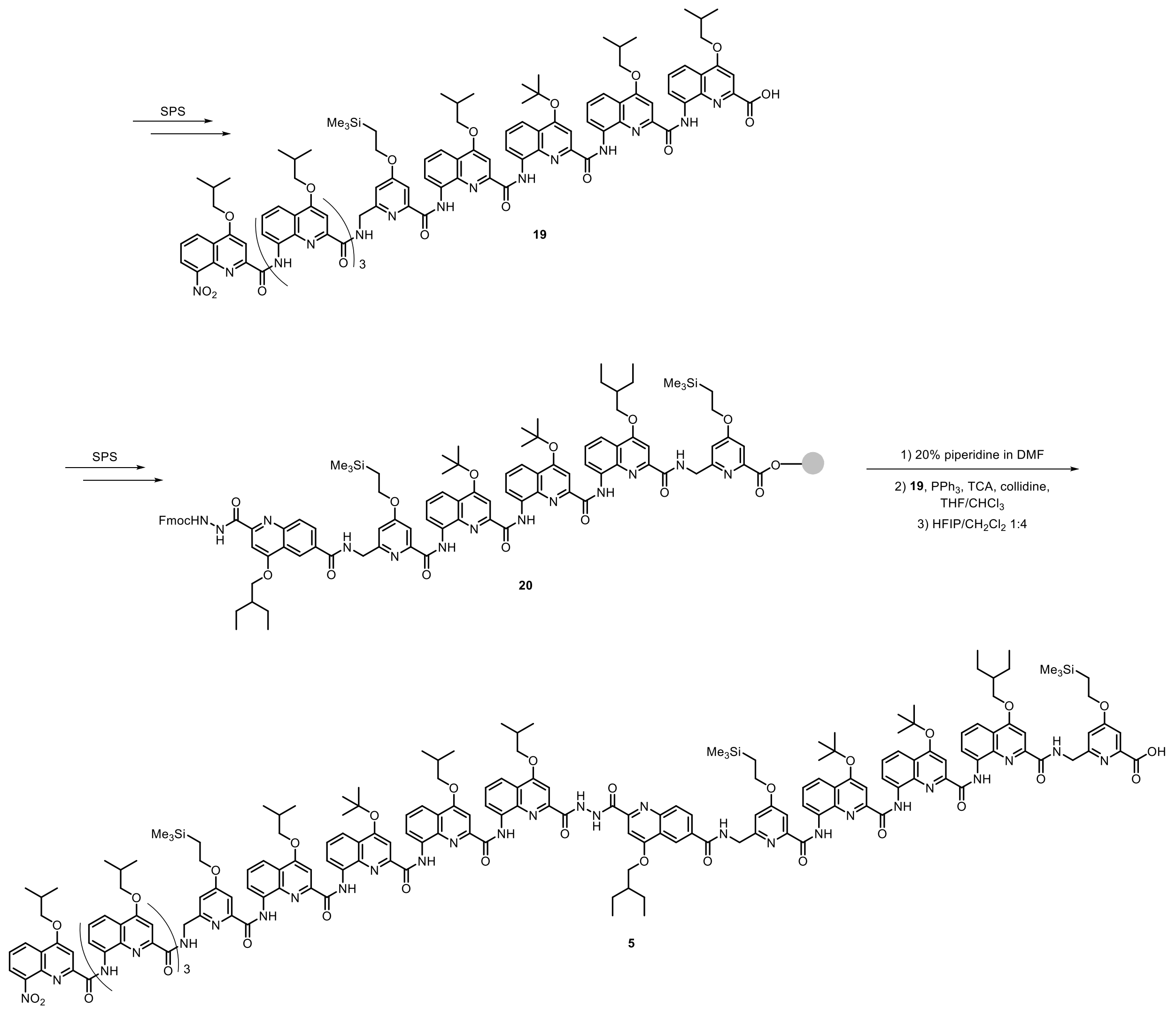

Scheme S3: Synthesis of **5**

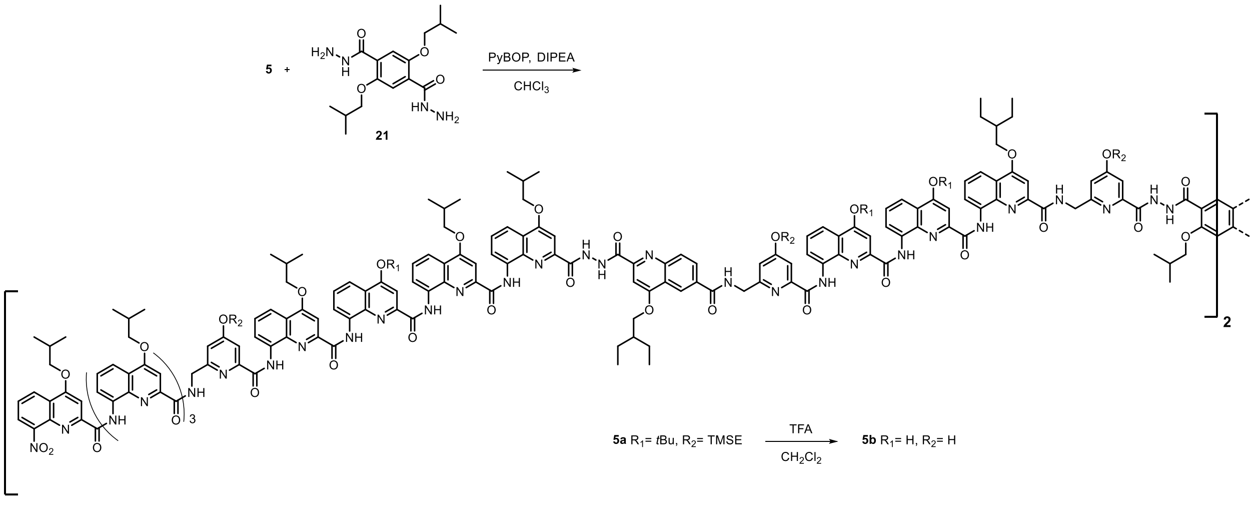

Scheme S4: Synthesis of **3b**

4. Experimental procedures

4.1 General methods

Commercial reagents were purchased from Sigma-Aldrich, Alfa-Aesar or TCI and were used without further purification unless otherwise specified. SASRIN resin (100-200 mesh, loading 0.7-1.0 mmol/g) was purchased from Bachem. Tetrahydrofuran (THF), dichloromethane (DCM) and toluene were dried over alumina columns (MBRAUN SPS-800 solvent purification system); chloroform and diisopropylethylamine (DIPEA) were distilled over calcium hydride (CaH_2) prior to use. Reactions were monitored by thin layer chromatography (TLC) on Merck silica gel 60-F254 plates and observed under UV light. Column chromatography purifications were carried out on Merck GEDURAN Si60 (40-63 μm). Preparative recycling GPC (gel permeation chromatography) was carried out on JAIGEL 20*600 mm columns (Japan Analytical Industry) in chloroform containing 1% ethanol and 0.5% triethylamine as mobile phase, with a flow rate of 7.5 mL/min. Monitoring by UV detection was carried out at 254 nm, 280 nm, 300 nm and 360 nm.

Analytical RP-HPLC analyses were performed on an Ultimate 3000 HPLC System (ThermoFisher Scientific) using a Hypersil GOLD Phenyl column (4.6 x 100 mm, 5 μm, ThermoFisher Scientific). The mobile phase was composed of H_2O (solvent A) and CH_3CN (solvent B). Semipreparative purifications of oligomers were performed on an Ultimate 3000 HPLC System (ThermoFisher Scientific) using a Hypersil GOLD Phenyl column (10 x 250 mm, 5 μm, ThermoFisher Scientific).

Solid phase synthesis (SPS) was performed manually under microwave irradiation on a CEM Discover (Liberty Bio) microwave oven using open reaction vessel and an internal fiber optic probe for temperature control.

High-resolution electrospray mass spectra were recorded on a Thermo Exactive orbitrap instrument.

4.2 Synthesis of small molecules

Compound 6. 4-amino benzoic acid (10 g, 72.9 mmol, 1 equiv.) was dissolved in MeOH (100 mL) under N_2. Then dimethyl acetylene-dicarboxylate (8.96 mL, 72.9 mmol, 1 equiv.) was added and the solution stirred at r.t. for 4 days. The solid obtained was collected by filtration, washed with MeOH and dried under vacuum. The compound was obtained as a light yellow solid (14.2 g, 79% yield).

^{1}H NMR (300 MHz, DMSO-d_6) δ 12.71 (s, 1H), 9.67 (s, 1H), 7.84 (d, J = 8.6 Hz, 2H), 6.98 (d, J = 8.6 Hz, 2H), 5.50 (s, 1H), 3.71 (s, 3H), 3.67 (s, 3H). ^{13}C NMR (75 MHz, DMSO-d_6) δ 167.68, 166.95, 164.36, 145.16, 144.59, 130.66, 124.93, 118.65, 97.60, 53.28, 51.39. MS calcd for $C_{13}H_{14}NO_6$ $[M+H]^+$ 280.0816, found (HR-ESI) 280.0815.

Compound 7. Diphenyl ether (50 mL) was heated up to its boiling point, then compound **6** (5.5 g, 19.7 mmol) was added as a solid. The reaction mixture was boiled for 12 min. After cooling to r.t., cyclohexane was added and the precipitate was filtered. The solid was washed thoroughly with cyclohexane and diethyl ether. After drying under vacuum, the compound was obtained as a brownish solid (4.1 g, 84% yield).

^{1}H NMR (500 MHz, DMSO-d_6) δ 13.10 (s, 1H), 12.30 (s, 1H), 8.66 (d, J = 1.5 Hz, 1H), 8.17 (dd, J = 8.8, 2.0 Hz, 1H), 7.99 (d, J = 8.8 Hz, 1H), 6.68 (s, 1H), 3.97 (s, 3H). ^{13}C NMR (126 MHz, DMSO-d_6) δ 177.62, 166.66, 162.45, 142.68, 138.39, 132.47, 127.11, 125.96, 125.14, 119.94, 111.17, 53.65. MS calcd for $C_{12}H_{10}NO_5$ $[M+H]^+$ 248.0553, found (HR-ESI) 248.0554.

Compound 8. Compound **7** (3 g, 12.1 mmol, 1 equiv.) and HBTU (4.6 g, 12.1 mmol, 1 equiv.) were dissolved in dry DMF (40 mL) under N_2. 2-(Trimethylsilyl)ethanol (2.6 mL, 18.2 mmol, 1.5 equiv.) and triethylamine (3.4 mL, 24.3 mmol, 2 equiv.) were added and the reaction mixture was stirred at r.t. for 48 h. The solvent was removed under vacuum and the residue suspended in CH_3CN. The precipitate was collected by filtration and washed thoroughly with CH_3CN and MeOH. After drying under vacuum, the compound was obtained as a light yellow solid (3.6 g, 85% yield).

^{1}H NMR (300 MHz, $CDCl_3$) δ 9.02 (d, J = 1.9 Hz, 1H), 8.30 (dd, J = 8.7, 2.0 Hz, 1H), 7.44 (d, J = 8.7 Hz, 1H), 6.99 (d, J = 1.8 Hz, 2H), 4.56 – 4.34 (m, 1H), 4.05 (s, 3H), 1.22 – 1.10 (m, 2H), 0.09 (s, J = 3.3 Hz, 9H). ^{13}C NMR (75 MHz, $CDCl_3$) δ 179.55, 165.95, 163.36, 141.72, 136.62, 133.67, 129.04, 126.96, 125.82, 118.31, 112.87, 63.85, 54.16, 17.66, -1.32. MS calcd for $C_{17}H_{22}NO_5Si$ $[M+H]^+$ 348.1261, found (HR-ESI) 348.1259.

Compound 9. Compound **8** (5 g, 14.4 mmol, 1.0 equiv.) and PPh_3 (4.15 g, 15.8 mmol, 1.1 equiv.) were dissolved in dry THF (100 mL) under N_2. Then 2-ethyl-1-butanol (1.95 mL, 15.8 mmol, 1.1 equiv.) was added and the reaction mixture was cooled to 0 °C. Diisopropylazodicarboxylate (DIAD) (3.1 mL, 15.8 mmol, 1.1 equiv.) was slowly added dropwise. The reaction mixture was allowed to come to r.t. and then stirred overnight. The solvent was evaporated, the residue was triturated in hexane and filtered. The filtrated was concentrated under vacuum and the residue purified by flash chromatography (300:0.5 CH_2Cl_2/MeOH). The product was obtained as a yellow solid (5.6 g, 90% yield).

^{1}H NMR (300 MHz, $CDCl_3$) δ 8.95 (d, *J* = 1.5 Hz, 1H), 8.33 (dd, *J* = 8.9, 1.9 Hz, 1H), 8.24 (dd, *J* = 8.9, 0.4 Hz, 1H), 7.61 (s, 1H), 4.50 (ddd, *J* = 16.9, 10.5, 6.9 Hz, 2H), 4.21 (d, *J* = 5.7 Hz, 2H), 4.08 (s, 3H), 1.99 – 1.81 (m, 1H), 1.68 – 1.52 (m, 5H), 1.23 – 1.14 (m, 2H), 1.00 (t, *J* = 7.5 Hz, 6H), 0.11 (s, *J* = 3.3 Hz, 9H). ^{13}C NMR (75 MHz, $CDCl_3$) δ 166.32, 166.22, 164.10, 151.10, 150.47, 130.54, 130.11, 129.42, 125.06, 121.93, 101.49, 71.62, 63.91, 53.56, 40.79, 23.64, 17.50, 11.29, -1.24. MS calcd for $C_{23}H_{34}NO_5Si$ $[M+H]^+$ 432.2201, found (HR-ESI) 432.2198.

Compound 10. Compound **9** (1.38 g, 3.2 mmol, 1 equiv.) was dissolved in a mixture of THF/H_2O 3:1 (20 mL) and $LiOH{\cdot}H_2O$ (147 mg, 3.5, 1.1 equiv.) was added. The reaction mixture was stirred at r.t. for 15 min. The solution was diluted with water and neutralized with an aqueous 5% citric acid solution. The precipitate obtained was recovered by filtration and washed thoroughly with H_2O. After drying under vacuum, the compound was obtained as a light yellow solid (1.28 g, 96% yield).

^{1}H NMR (300 MHz, $CDCl_3$) δ 8.98 (d, *J* = 1.6 Hz, 1H), 8.39 (dd, *J* = 8.8, 1.8 Hz, 1H), 8.15 (d, *J* = 8.8 Hz, 1H), 7.70 (s, 1H), 4.59 – 4.46 (m, 2H), 4.26 (d, *J* = 5.7 Hz, 2H), 1.97-1.89 (m, 1H), 1.68 – 1.53 (m, 4H), 1.23 – 1.14 (m, 2H), 1.01 (t, *J* = 7.5 Hz, 6H), 0.12 (s, 9H). ^{13}C NMR (75 MHz, $CDCl_3$) δ 166.11, 165.92, 163.85, 150.18, 147.39, 131.39, 130.01, 128.27, 125.34, 122.02, 100.02, 72.52, 64.20, 40.67, 23.55, 17.48, 11.23, -1.23. MS calcd for $C_{22}H_{32}NO_5Si$ $[M+H]^+$ 418.2044, found (HR-ESI) 418.2042.

Compound 11. Compound **10** (2.6 g, 6.2 mmol, 1 equiv.) and HBTU (2.36 g, 6.2 mmol, 1 equiv.) were dissolved in dry DMF under N_2. Then 9-fluorenylmethyl carbazate (2.37 g, 9.3 mmol, 1.5 equiv.) and DIPEA (2.2 mL, 12.6 mmol, 2 equiv.) were added. The solution was stirred at r.t for 48 h. After removing the solvent under vacuum, the crude was purified by flash chromatography (8:2 cyclohexane/EtOAc). The product was obtained as a yellow solid (3.1 g, 75% yield).

^1H NMR (300 MHz, DMSO-d_6) δ 10.70 (s, 1H), 9.55 (s, 1H), 8.84 (s, 1H), 8.31 (d, *J* = 8.9 Hz, 1H), 8.15 (d, *J* = 8.9 Hz, 1H), 7.92 (d, *J* = 7.2 Hz, 2H), 7.78 (d, *J* = 7.2 Hz, 2H), 7.65 (s, 1H), 7.47-7.34 (m, 4H), 4.49 (t, *J* = 7.9 Hz, 2H), 4.42-4.40 (m, 3H), 4.32 (d, *J* = 5.5 Hz, 2H), 1.89-1.81 (m, 1H), 1.60-1.50 (m, 4H), 1.14 (t, *J* = 7.8 Hz, 2H), 0.97 (t, *J* = 7.4 Hz, 6H), 0.09 (s, 9H) (mixture of two conformers, only the major peaks are reported). ^{13}C NMR (75 MHz, $CDCl_3$) δ 166.32, 164.34, 163.77, 156.12, 151.43, 149.59, 143.59, 141.38, 130.17, 129.66, 129.11, 127.89, 127.23, 125.25, 121.98, 120.10, 99.32, 71.73, 68.20, 63.93, 47.01, 40.69, 23.56, 17.46, 11.26, -1.23. MS calcd for $C_{37}H_{44}N_3O_6Si$ $[M+H]^+$ 654.2994, found (HR-ESI) 654.2998.

Compound 12. Compound **11** (2 g, 3 mmol) was treated with a solution of TFA 50% in CH_2Cl_2 (15 mL) at r.t. under stirring for 24 h. The solvent was removed under vacuum, the residue was triturated in Et_2O, filtered and dried. The compound was obtained as a white solid (1.5 g, 89% yield).

^1H NMR (300 MHz, DMSO-d_6) δ 13.38 (s, 1H), 10.69 (s, 1H), 9.54 (s, 1H), 8.82 (s, 1H), 8.30 (d, *J* = 8.6 Hz, 1H), 8.13 (d, *J* = 8.8 Hz, 1H), 7.92 (d, *J* = 7.4 Hz, 2H), 7.78 (d, *J* = 7.1 Hz, 2H), 7.64 (s, 1H), 7.47-7.34 (m, 4H), 7.03 (m, 1H), 4.41 (m, 2H), 4.32 (m, 3H), 1.92 – 1.78 (m, 1H), 1.59-1.50 (m, 4H), 0.96 (t, *J* = 7.4 Hz, 6H) (mixture of two conformers, only the major peaks are reported). ^{13}C NMR (75 MHz, DMSO-d_6) δ 166.86, 163.83, 163.38, 156.14, 152.82, 149.10, 143.77, 140.89, 130.20, 129.76, 129.25, 127.87, 127.30, 125.43, 124.22, 121.04, 120.30, 99.63, 71.20, 66.35, 65.06, 46.63, 23.10, 15.28, 11.09. MS calcd for $C_{32}H_{32}N_3O_6$ $[M+H]^+$ 554.2286, found (HR-ESI) 554.2295.

Compound 15. Compound **13**[5] (170 mg. 0.63 mmol, 1.03 equiv.), compound **14**[6] (300 mg, 0.61 mmol, 1 equiv.) and PyBOP (656 g, 1.26 mmol, 2 equiv.) were dissolved in dry $CHCl_3$ (15 mL) under N_2. DIPEA (450 µL, 2.52 mmol, 4 equiv.) was added and the solution stirred at r.t. for 2 days. The reaction mixture was diluted with CH_2Cl_2, washed with citric acid solution 5% and $NaHCO_3$ 5% solution. The organic phase was dried over $MgSO_4$, filtered and concentrated. The crude was purified by chromatography using CH_2Cl_2. The compound was obtained as a yellow solid (300 mg, 66% yield).

^1H NMR (500 MHz, $CDCl_3$) δ 12.77 (s, 1H), 8.95 (d, *J* = 7.6 Hz, 1H), 7.93 (d, *J* = 8.5 Hz, 1H), 7.75 (s, 1H), 7.72 (d, *J* = 2.3 Hz, 1H), 7.65 (t, *J* = 8.1 Hz, 1H), 7.57 (d, *J* = 7.6 Hz, 2H), 7.46 (d, *J* = 7.5 Hz, 2H), 7.21 (t, *J* = 7.4 Hz, 2H), 6.98 – 6.92 (m, 3H), 6.84 (t, *J* = 6.3 Hz, 1H), 4.61 (d, *J* = 6.3 Hz, 2H), 4.36 (d, *J* = 7.1 Hz, 2H), 4.24 (t, *J* = 8.0 Hz, 2H), 4.10 (t, *J* = 7.0 Hz, 1H), 4.03 (s, 3H), 1.69 (s, 9H), 1.19 (t, *J* = 8.3 Hz, 2H), 0.11 (s, 9H). ^{13}C NMR (126 MHz, $CDCl_3$) δ 167.36, 165.84, 162.43, 161.00, 158.43, 157.17, 151.82, 146.34, 143.97, 141.26, 140.18,

135.23, 128.51, 127.55, 126.82, 125.07, 124.76, 119.83, 117.17, 116.77, 111.36, 106.98, 106.66, 81.76, 66.76, 66.58, 53.20, 47.34, 45.98, 28.80, 17.62, -1.20. MS calcd for $C_{42}H_{47}N_4O_7Si$ $[M+H]^+$ 747.3209, found (HR-ESI) 747.3208.

Compound 16. Compound **15** (300 mg, 0.4 mmol, 1 equiv.) was dissolved in degassed EtOAc (30 mL) under N_2. Then LiI (165 mg, 1.2 mmol, 3 equiv.) was added and the solution refluxed for 48 h under N_2 and in the dark. The solid precipitated from the reaction mixture was collected by centrifugation, washed with EtOAc (2v), 5% thiosulphate solution and water. The product was obtained after drying as a white solid (205 mg, 85% yield).

^{1}H NMR (500 MHz, DMSO-d_6) δ 13.40 (s, 1H), 9.44 (t, J = 6.5 Hz, 1H), 8.60 (dd, J = 7.5, 1.3 Hz, 1H), 7.89 (s, 1H), 7.79 (dd, J = 8.5, 1.4 Hz, 1H), 7.75 (d, J = 7.5 Hz, 2H), 7.59 (d, J = 7.2 Hz, 2H), 7.53 (t, J = 8.0 Hz, 1H), 7.50 (d, J = 2.2 Hz, 1H), 7.22 (t, J = 7.5 Hz, 2H), 7.15 (d, J = 2.3 Hz, 1H), 6.85 (td, J = 7.4, 1.1 Hz, 2H), 4.42 (d, J = 6.5 Hz, 2H), 4.31 – 4.26 (m, 2H), 4.06 – 4.03 (m, 2H), 4.01-3.98 (m, 1H), 1.59 (s, 9H), 1.18 – 1.11 (m, 2H), 0.09 (s, 9H). ^{13}C NMR (126 MHz, DMSO-d_6) δ 166.95, 166.61, 161.28, 160.38, 158.73, 158.14, 156.84, 150.87, 143.72, 140.43, 139.25, 134.35, 128.92, 127.32, 126.78, 125.64, 122.94, 121.38, 120.03, 119.78, 116.16, 114.32, 110.62, 108.34, 106.02, 80.29, 66.20, 65.75, 46.48, 45.30, 28.26, 16.91, -1.31. MS calcd for $C_{41}H_{45}N_4O_7Si$ $[M+H]^+$ 733.3052, found (HR-ESI) 733.3055.

4.3 Solid phase synthesis general methods

The oligomers **17-20** were synthesized using SPS on SASRIN resin using previously reported procedures[6,7]

Quinoline monomers (Fmoc-Q-OH[7], Fmoc-X-OH[6], Fmoc-Q^h-OH[8] and **12**) were activated *via* formation of acid chloride. Dimeric blocks Fmoc-YQ-OH[6] and **16** were first prepared in solution and then coupled on solid support following standard activation and coupling conditions.

Oligomers **18** and **20** have not been cleaved from resin after SPS, indeed solid-phase fragment condensation was performed directly to obtain compounds **4** and **5**.

4.4 Synthesis of oligomers

O_2N-QQQQYQXQQ-OH (**19**). Compound **19** was synthesized using the SPS procedures previously described. After cleavage from the resin, the crude product was purified by semi-preparative RP-HPLC. After lyophilization, the product was recovered as a yellow solid.

^{1}H NMR (500 MHz, $CDCl_3$) δ 11.37 (s, 1H), 11.35 (s, 1H), 11.01 (s, 1H), 10.92 (s, 1H), 10.85 (s, 1H), 10.74 (s, 1H), 10.53 (s, 1H), 8.30 (dd, *J* = 8.1, 1.5 Hz, 1H), 8.29 (dd, *J* = 7.5, 1.2 Hz, 1H), 8.23-8.21 (m, 1H), 8.20 (dd, *J* = 7.5, 1.2 Hz, 1H), 8.07 (dd, *J* = 7.6, 1.3 Hz, 1H), 7.97 (dd, *J* = 8.2, 1.3 Hz, 1H), 7.93 (dd, *J* = 7.5, 1.3 Hz, 1H), 7.89 (dd, *J* = 8.2, 1.3 Hz, 1H), 7.88 (dd, *J* = 8.3, 1.2 Hz, 1H), 7.80 (dd, *J* = 8.2, 1.3 Hz, 1H), 7.77 (d *J* = 8.4 Hz, 2H), 7.71 (dd, *J* = 8.3, 1.3 Hz, 1H), 7.66 (s, 1H), 7.55 (s, 1H), 7.51 – 7.46 (m, 2H), 7.47 (s, 1H), 7.38 (t, *J* = 7.9 Hz, 1H), 7.36 – 7.32 (m, 1H), 7.31 – 7.28 (m, 2H), 7.25-7.22 (m, 1H), 7.16 – 7.09 (m, 1H), 7.12 (s, 1H), 7.10 – 7.06 (m, 2H), 7.04 (t, *J* = 7.8 Hz, 1H), 6.89 (t, *J* = 7.9 Hz, 1H), 6.72 (d, *J* = 2.3 Hz, 1H), 6.65 (s, 1H), 6.53 (d, *J* = 2.3 Hz, 1H), 6.49 (s, 1H), 6.39 (s, 1H), 5.95 (s, 1H), 5.80 (s, 1H), 4.35-4.26 (m, 2H), 4.16 (dd, *J* = 8.5, 5.8 Hz, 1H), 4.09 (dd, *J* = 8.4, 6.2 Hz, 1H), 4.03-3.89 (m, 4H), 3.85-3.76 (m, 4H), 3.72 (d, *J* = 6.4 Hz, 2H), 3.67 – 3.63 (m, 1H), 3.56 – 3.51 (m, 1H), 3.42 (dd, *J* = 17.0, 3.6 Hz, 1H), 2.48-2.41 (m, 2H), 2.38-2.18 (m, 5H), 1.41 (t, *J* = 8.9 Hz, 1H), 1.31 (d, *J* = 4.0 Hz, 3H), 1.30 (d, *J* = 4.0 Hz, 3H), 1.26-1.25 (m, 3H), 1.25 (s, 9H), 1.23 (d, *J* = 6.7 Hz, 3H), 1.20 (d, *J* = 6.7 Hz, 6H), 1.18 (d, *J* = 6.7 Hz, 6H), 1.15 (d, *J* = 6.6 Hz, 6H), 1.14 (d, *J* = 6.7 Hz, 6H), 1.12 (d, *J* = 6.7 Hz, 3H), 1.09 (d, *J* = 6.7 Hz, 3H), 0.91 – 0.86 (m, 2H), 0.32 (s, 9H). MS calcd for $C_{124}H_{132}N_{18}O_{21}Si$ $[M+2H]^{2+}$ 1118.4792, found (HR-ESI) 1118.4780.

O_2N-QQQQYQXQQ-T2-YXXQ^hY-OH (**5**). Fmoc-T2-YXXQ^hY-Resin (**20**) (5.36 µmol, 1 equiv.) was obtained using standard SPS protocol. After Fmoc deprotection (20% piperidine in DMF, 2v x 10 min) and washing with DMF and dry THF, the resin was suspended in dry

THF (500 µL), to which was added collidine (4 µL, 30.5 µmol, 5.7 equiv). Then a solution of **19** (24 mg, 10.7 µmol, 2 equiv.), PPh_3 (8 mg, 30.5 µmol, 5.7 equiv) and TCA (3 µL, 30.5 µmol, 5.7 equiv) in dry $CHCl_3$ (500 µL) was added to resin. The reaction was carried out under MW irradiation (25 W) at 50 °C for 15 min. The resin was washed briefly with dry THF, and the process repeated. Successively the resin was washed with DMF and CH_2Cl_2, and dried under vacuum. The compound was cleaved from the resin using HFIP/CH_2Cl_2 3:7 for 1 h r.t (2v). The solvent was removed under reduced pressure. The pure product was obtained by precipitation from CH_2Cl_2/MeOH as a yellow solid (17 mg, 83% yield).

^{1}H NMR (500 MHz, $CDCl_3$) δ 11.71 (s, 1H), 11.69 (s, 1H), 11.66 (s, 1H), 11.42 (s, 1H), 11.28 (s, 1H), 11.06 (s, 1H), 10.96 (s, 1H), 10.87 (s, 1H), 10.82 (s, 1H), 10.46 (s, 1H), 10.10 (d, *J* = 8.0 Hz, 1H), 9.74 (d, *J* = 8.0 Hz, 1H), 8.44 (d, *J* = 7.4 Hz, 1H), 8.28 (d, *J* = 7.9 Hz, 1H), 8.20 (d, *J* = 7.2 Hz, 1H), 8.09 (s, 2H), 8.05 (dd, *J* = 7.4, 1.4 Hz, 2H), 8.02 – 7.89 (m, 8H), 7.84-7.77 (m, 4H), 7.73 (t, *J* = 6.6 Hz, 2H), 7.69 (s, 1H), 7.68 (dd, *J* = 7.8, 1.2 Hz, 1H), 7.63 – 7.57 (m, 2H), 7.48-7.46 (m, 2H), 7.39 (t, *J* = 7.9 Hz, 2H), 7.34 (s, 1H), 7.31-7.19 (m, overlap with solvent pick), 7.12 – 7.05 (m, 5H), 7.03 (t, *J* = 7.7 Hz, 1H), 6.90 (t, *J* = 7.7 Hz, 1H), 6.84 (s, 1H), 6.70 (d, *J* = 2.2 Hz, 1H), 6.68 (s, 1H), 6.63 (s, 1H), 6.61 (d, *J* = 1.5 Hz, 1H), 6.56 (s, 1H), 6.51 (s, 1H), 6.49 (d, *J* = 2.2 Hz, 1H), 6.48 (d, *J* = 2.2 Hz, 1H), 6.46 (s, 1H), 6.35 (d, *J* = 1.5 Hz, 1H), 5.91 (s, 1H), 5.76 (s, 1H), 4.38-4-34 (m, 2H), 4.22 – 4.12 (m, 2H), 4.11 – 4.00 (m, 6H), 3.97-3.85 (m, 8H), 3.83-3.73 (m, 6H), 3.64 (t, *J* = 7.6 Hz, 2H), 3.50-3.44 (m, 2H), 3.31-3.22 (m, 2H), 3.13 (t, *J* = 8.4 Hz, 2H), 2.50-2.41 (m, 3H), 2.37-2.19 (m, 6H), 1.85 (s, 9H), 1.81 (s, 9H), 1.38-1.34 (m, 2H), 1.33 (d, *J* = 6.8 Hz, 3H), 1.32 (d, *J* = 6.7 Hz, 3H), 1.28 (s, 9H), 1.26 (d, *J* = 6.8 Hz, 3H), 1.24 (d, *J* = 6.8 Hz, 3H), 1.21 – 1.13 (m, 18H), 0.94-0.73 (m, 10H), 0.56 (t, *J* = 7.4 Hz, 3H), 0.45 (t, *J* = 7.4 Hz, 3H), 0.23 (s, 9H), 0.07 (s, 9H), 0.06 (s, 9H), (mixture of two conformers, only the major peaks are reported). MS calcd for $C_{209}H_{233}N_{31}O_{34}Si_3$ $[M+2H]^{2+}$ 1902.3382, found (HR-ESI) 1902.3469.

O_2N-Q<u>X</u>QQ<u>Y</u>Q<u>X</u>QQ-T2-Q<u>X</u>QQ<u>Y</u>Q<u>X</u>QQ-OMe (2a). Fmoc-T2-Q<u>X</u>QQ<u>Y</u>Q<u>X</u>QQ-Resin (**18**) (6.75 µmol, 1 equiv.) was obtained using standard SPS protocol. After Fmoc deprotection (20% piperidine in DMF, 2v x 10 min) and washing with DMF and dry THF, the resin was suspended in dry THF (500 µL), to which was added collidine (6 µL, 38.5 µmol, 5.7 equiv). Then a solution of **17**[6] (30 mg, 13.4 µmol, 2 equiv.), PPh_3 (10 mg, 38.5 µmol, 5.7 equiv) and TCA (4.6 µL, 38.5 µmol, 5.7 equiv) in dry $CHCl_3$ (500 µL) was added to resin. The reaction was carried out under MW irradiation (25 W) at 50 °C for 15 min. The resin was washed briefly with dry THF, and the process repeated. Successively the resin was washed with DMF and

CH_2Cl_2, and dried under vacuum. The compound was cleaved from the resin using 1% TFA in CH_2Cl_2. After removal of the solvent, compound **4** (O_2N-QXQQYQXQQ-T2-QXQQYQXQQ-OH) was obtained and used without any additional purification.

Crude compound **4** (20 mg, 4.22 μmol, 1 equiv.) was dissolved in a mixture of dry THF/MeOH 3:2 (1.25 mL) under N_2. $TMSCHN_2$ (soluz. 2M in hexane, 5 μL, 8.44 μmol, 2 equiv.) was added dropwise and the solution stirred at r.t. for 2h. Few drops of acetic acid were added and the solution stirred for 5 min at r.t. Then the solution was diluted with CH_2Cl_2, washed with $NaHCO_3$, dried $MgSO_4$, filtered and concentrated. The pure compound was obtained by precipitation from CH_2Cl_2/MeOH as a yellow solid (15 mg, 75% yield).

^{1}H NMR (500 MHz, $CDCl_3$) δ 11.43 (s, 1H), 11.34 (s, 1H), 11.28 (s, 1H), 11.23 (s, 1H), 11.18 (s, 1H), 11.05 (s, 1H), 10.97 (s, 1H), 10.89 (s, 1H), 10.75 (s, 2H), 10.73 (s, 1H), 10.65 (s, 1H), 10.33 (s, 2H), 10.00 (d, *J* = 8.7 Hz, 1H), 9.84 (s, 1H), 9.65 (d, *J* = 7.5 Hz, 1H), 8.26 (dd, *J* = 7.9, 1.5 Hz, 1H), 8.18 (dd, *J* = 7.2, 1.0 Hz, 1H), 8.08 (d, *J* = 7.5 Hz, 1H), 8.04 (d, *J* = 6.9 Hz, 1H), 8.01 – 7.97 (m, 2H), 7.96 – 7.90 (m, 3H), 7.89-7.84 (m, 3H), 7.83- 7.80 (m, 3H), 7.78-7.70 (m, 5H), 7.69-7.62 (m, 4H), 7.59 (s, 1H), 7.56-7.62 (m, 1H), 7.52 – 7.44 (m, 1H), 7.40 – 7.37 (m, 1H), 7.33-7.27 (m, 5H), 7.19 (dd, *J* = 7.2, 1.4 Hz, 1H), 7.16 (t, *J* = 7.7 Hz, 1H), 7.09 – 7.04 (m, 4H), 7.02 (t, *J* = 7.6 Hz, 2H), 7.00-6.95 (m, 2H), 6.93 (t, *J* = 7.7 Hz, 1H), 6.89 (t, *J* = 7.7 Hz, 1H), 6.78 (t, *J* = 7.6 Hz, 1H), 6.62 (s, 1H), 6.56 (s, 1H), 6.54 (s, 1H), 6.49 (s, 1H), 6.46 – 6.44 (m, 1H), 6.44 (s, 1H), 6.39-6.37 (m, 1H), 6.36 (s, 1H), 6.31 (s, 1H), 6.27 (s, 2H), 6.19 (s, 1H), 5.88 (s, 1H), 5.74 (s, 1H), 5.73 (s, 1H), 5.71 (s, 1H), 4.21 – 3.99 (m, 8H), 3.98-3.89 (m, 6H), 3.84-3.72 (m, 6H), 3.69-3.64 (m, 2H), 3.58 (t, *J* = 6.5 Hz, 3H), 3.54 (t, *J* = 7.5 Hz, 1H), 3.46 (t, *J* = 7.4 Hz, 1H), 3.21 – 3.16 (m, 1H), 3.06 (s, 3H), 2.86 – 2.81 (m, 1H), 2.78 (t, *J* = 8.4 Hz, 1H), 2.49 – 2.20 (m, 13H), 2.15-2.06 (m, 2H), 1.90 – 1.84 (m, 2H), 1.81 (s, 9H), 1.63 (s, 9H), 1.59 (d, *J* = 2.5 Hz, 2H), 1.58 (s, 9H), 1.32 – 1.12 (m, 69H), 1.06 (s, 9H), 1.03 (d, *J* = 6.8 Hz, 3H), 0.73 (p, *J* = 7.4 Hz, 2H), 0.61 (p, *J* = 7.4, 6.9 Hz, 2H), 0.45 (t, *J* = 7.4 Hz, 3H), 0.28 (s, 9H), 0.22 (t, *J* = 7.5 Hz, 3H), -0.06 (s, 9H) (mixture of two conformers, only the major peaks are reported). MS calcd for $C_{266}H_{283}N_{39}O_{42}Si_2$ $[M+2H]^{2+}$ 2375.5368, found (HR-ESI) 2375.5598.

O_2N-QXQQYQXQQ-T2-QXQQYQXQQ-OMe (**2b**). Compound **2a** (5 mg, 1 μmol) was dissolved in a solution of 50% TFA in CH_2Cl_2 (1 mL). The solution was stirred at r.t. for 4h. Then the solvent was removed under reduced pressure. The residue was suspended in CH_3CN/water and freeze-dried. The product was obtained as a yellow solid (quantitative yield).

^{1}H NMR (500 MHz, $CDCl_3$) δ 12.06 (s, 1H), 11.95 (s, 1H), 11.78 (s, 1H), 11.57 (s, 1H), 11.52 (s, 1H), 11.44 (s, 1H), 11.32 (s, 1H), 11.23 (s, 1H), 11.22 (s, 1H), 11.17 (s, 1H), 10.96 (s, 1H), 10.46 (d, *J* = 8.8 Hz, 1H), 10.39 (s, 1H), 10.37 (s, 1H), 10.35 (s, 1H), 10.24 (s, 1H), 10.17 (s, 1H), 10.13 (s, 1H), 9.42 (s, 1H), 9.10 (d, *J* = 8.8 Hz, 1H), 9.08 (s, 1H), 8.62 (d, *J* = 7.1 Hz, 1H), 8.44-8.34 (m, 2H), 8.27 (d, *J* = 7.2 Hz, 1H), 8.19 (d, *J* = 8.1 Hz, 1H), 8.17 – 8.08 (m, 6H), 8.06 (dd, *J* = 8.1, 1.0 Hz, 2H), 7.94-7.87 (m, 4H), 7.85 (d, *J* = 8.4 Hz, 1H), 7.81 (dd, *J* = 8.0, 0.9 Hz, 1H), 7.78 – 7.74 (m, 2H), 7.70 – 7.56 (m, 5H), 7.56 – 7.49 (m, 4H), 7.43 – 6.98 (m, overlap with solvent peak), 6.92 (t, *J* = 7.7 Hz, 1H), 6.84-6.82 (m, 1H), 6.81-6.79 (m, 1H), 6.77 (s, 1H), 6.76 (d, *J* = 7.2 Hz, 1H), 6.71 (s, 1H), 6.65 (d, *J* = 7.0 Hz, 1H), 6.55 (s, 1H), 6.47 (s, 1H), 6.42 (s, 1H), 6.13 (s, 1H), 6.12 (s, 1H), 5.39 (s, 1H), 5.31 (s, 1H), 4.28-4.21 (m, 2H), 4.15 (t, *J* = 6.6 Hz, 2H), 4.11-4.04 (m, 2H), 4.03-3.92 (m, 4H), 3.91– 3.87 (m, 4H), 3.82-3.77 (m, 6H), 3.69-3.63 (m, 6H), 3.41 (t, *J* = 6.4 Hz, 1H), 3.30-3.28 (m, 1H), 3.27 (s, 3H), 2.89 (t, *J* = 8.6 Hz, 1H), 2.79-2.74 (m, 1H), 2.59 – 2.40 (m, 6H), 2.37-2.13 (m, 7H), 1.60-1.50 (m, 4H), 1.45-1.40 (m, 12H), 1.39-1.35 (m, 6H), 1.34-1.30 (m, 6H), 1.24-1.18 (m, 36H), 1.15-1.09 (m, 12H) 0.33 (t, *J* = 7.5 Hz, 3H), 0.14 (t, *J* = 7.4 Hz, 3H). MS calcd for $C_{240}H_{227}N_{39}O_{42}$ $[M+2H]^{2+}$ 2163.3407, found (HR-ESI) 2163.3641.

(O_2N-QQQQ<u>Y</u>Q<u>X</u>QQ-T2-<u>YXX</u>Q^h<u>Y</u>)$_2$-T1 (**3a**). Compounds **5** (22 mg, 5.78 µmol, 1 equiv.), **21**[5] (0.98 mg, 2.89 µmol, 0.5 equiv.), PyBOP (6 mg, 17.3 µmol, 3 equiv.) and DIPEA (5 µL, 28 µmol, 5 equiv.) were dissolved in dry CH_2Cl_2 (500 µL). The reaction mixture was stirred at r.t. under N_2 for 72 h. The product was obtained after GPC purification as a yellow solid (10 mg, 44% yield).

^{1}H NMR (500 MHz, $CDCl_3$) δ 11.92 (s, 2H), 11.73 (s, 2H), 11.64 (s, 2H), 11.40 (s, 2H), 11.27 (s, 2H), 11.06 (s, 2H), 10.96 (s, 2H), 10.86 (s, 2H), 10.82 (s, 2H), 10.45 (s, 2H), 10.36 (d, *J* = 8.8 Hz, 2H), 10.10 (s br, 2H), 10.03 (d, *J* = 8.9 Hz, 2H), 9.74 (d, *J* = 7.5 Hz, 2H), 8.34 (s br, 2H), 8.31 – 8.23 (m, 6H), 8.18 (d, *J* = 6.9 Hz, 2H), 8.08 (s br, 2H), 8.05 (d, *J* = 7.2 Hz, 4H), 8.00 – 7.89 (m, 10H), 7.88 – 7.83 (m, 2H), 7.83 – 7.76 (m, 6H), 7.71 (d, *J* = 7.0 Hz, 2H), 7.67 (dd, *J* = 8.0, 1.2 Hz, 2H), 7.62 (d, *J* = 8.0 Hz, 2H), 7.52 – 7.44 (m, 8H), 7.39 (d, *J* = 1.9 Hz, 2H), 7.34 – 7.30 (m, 4H), 7.25 – 7.22 (m, overlap with solvent), 7.17 (s, 2H), 7.12 – 7.04 (m, 8H), 7.02 (t, *J* = 7.5 Hz, 2H), 6.90 (t, *J* = 7.7 Hz, 2H), 6.79 (s, 2H), 6.72 – 6.65 (m, 6H), 6.56 (s, 2H), 6.48-6.45 (m, 6H), 6.42 (d, *J* = 1.4 Hz, 2H), 6.33 (d, *J* = 2.0 Hz, 2H), 5.91 (s, 2H), 5.75 (s, 2H), 4.35-4.29 (m, 2H), 4.22 – 4.14 (m, 4H), 4.11 – 4.03 (m, 10H), 4.03-3.84 (m, 22H), 3.83-3.71 (m, 10H), 3.66 – 3.62 (m, 4H), 3.56 (d, *J* = 14.8 Hz, 2H), 3.50 – 3.46 (m, 2H), 3.26 (d, *J* = 14.8 Hz, 2H), 3.07 (t, *J* = 8.2 Hz, 2H), 2.52-2.42 (m, 4H), 2.38-2.17 (m, 16H), 1.83 (s,

18H), 1.53 (s, 18H), 1.33 (d, J = 6.7 Hz, 6H), 1.32 (d, J = 6.7 Hz, 6H), 1.29 (s, 18H), 1.27 – 1.22 (m, 18H), 1.21 – 1.11 (m, 18H), 1.07 (m, 12H), 0.90 – 0.86 (m, 6H), 0.76 – 0.68 (m, 4H), 0.53 (t, J = 7.4 Hz, 6H), 0.39 (t, J = 7.4 Hz, 6H), 0.14 (s, 18H), 0.03 (s, 18H), 0.01 (s, 18H). (mixture of two conformers, only the major peaks are reported). MS calcd for $C_{434}H_{487}N_{66}O_{70}Si_6$ $[M+3H]^{3+}$ 2636.8392, found (HR-ESI) 2636.8266.

(O_2N-QQQQYQXQQ-T2-YXXQ^{h}Y)$_2$-T1 (**3b**). Compound **3a** (6 mg, 0.76 µmol) was dissolved in a solution of 50% TFA in CH_2Cl_2 (1 mL). The solution was stirred at r.t. for 24h. Then the solvent was removed under reduced pressure. The residue was suspended in CH_3CN/water and freeze-dried. The product was obtained as a yellow solid (quantitative yield). ^{1}H NMR (500 MHz, $CDCl_3$) δ 12.75 (s, 2H), 12.56 (s, 2H), 12.00 (s, 2H), 11.65 (s, 2H), 11.53 (s, 2H), 11.39 (s, 2H), 11.03 – 10.90 (m, 6H), 10.69 (d, J = 9.0 Hz, 2H), 10.61 (s, 2H), 10.58 (s, 2H), 10.53 (s, 2H), 10.32 (s, 2H), 10.11 (d, J = 9.1 Hz, 2H), 9.18 (d, J = 7.5 Hz, 2H), 9.06 (s br, 2H), 8.83 (d, J = 6.9 Hz, 2H), 8.76 (s br, 2H), 8.60 (d, J = 7.0 Hz, 2H), 8.55 (d, J = 7.8 Hz, 2H), 8.47 (d, J = 7.0 Hz, 2H), 8.43 – 8.37 (m, 4H), 8.32 (d, J = 7.5 Hz, 4H), 8.17-8.07 (m, 4H), 8.05 – 7.96 (m, 4H), 7.91 – 7.87 (m, 4H), 7.84 – 7.71 (m, 4H), 7.61 – 7.54 (m, 4H), 7.53-7.47 (m, 4H), 7.45 – 7.31 (m, 6H), 7.17 – 7.09 (m, 8H), 6.85 (d, J = 6.9 Hz, 2H), 6.45 (s, 2H), 5.99 (s, 2H), 5.70 (s, 2H), 4.54-4.50 (m, 2H), 4.36-4.26 (m, 4H), 4.14-4.07 (m, 8H), 4.02-3.87 (m, 12H), 3.85 – 3.79 (m, 8H), 3.54 – 3.48 (m, 4H), 3.39-3.30 (m, 4H), 3.25-3.18 (m, 4H), 2.97 – 2.87 (m, 4H), 2.79-2.74 (m, 2H), 2.65-2.58 (m, 2H), 2.54-2.45 (m, 6H), 2.41 – 2.28 (m, 12H), 2.27-2.22 (m, 6H), 1.52 (d, J = 6.7 Hz, 6H), 1.48 (d, J = 6.6 Hz, 6H), 1.43-1.17 (m, overlap with grease), 0.65 (d, J = 6.9 Hz, 6H), 0.45-0.41 (m, 12H), 0.32 (t, J = 7.5 Hz, 6H). MS calcd for $C_{380}H_{367}N_{66}O_{70}$ $[M+3H]^{3+}$ 2324.5723, found (HR-ESI) 2324.5744.

5. References

[1] Rigaku Oxford Diffraction (2015). *CrysAlis PRO*. Rigaku Oxford Diffraction, Yarnton, England.
[2] G. M. Sheldrick, *Acta Cryst.*, 2015, **A71**, 3-8.
[3] O.V. Dolomanov, L.J . Bourhis, R.J . Gildea, J.A.K. Howard, H. Puschmann, *J. Appl. Cryst.*, 2009, **42**, 339-341.
[4] G. M. Sheldrick, *Acta Cryst.*, 2015, **C71**, 3-8.
[5] S. De, B. Chi, T. Granier, T. Qi, V. Maurizot, I. Huc, *Nat. Chem.* **2018**, *10*, 51-57.
[6] D. Mazzier, S. De, B. Wicher, V. Maurizot, I. Huc, *Chem. Sci.* **2019**, *10*, 6984-6991.
[7] B. Baptiste, C. Douat-Casassus, K. Laxmi-Reddy, F. Godde, I. Huc, *J. Org. Chem.* **2010**, *75*, 7175-7185.
[8] The synthesis of the **Q^h** monomer will be reported elsewhere.

6. ^{1}H and ^{13}C NMR spectra of new compounds

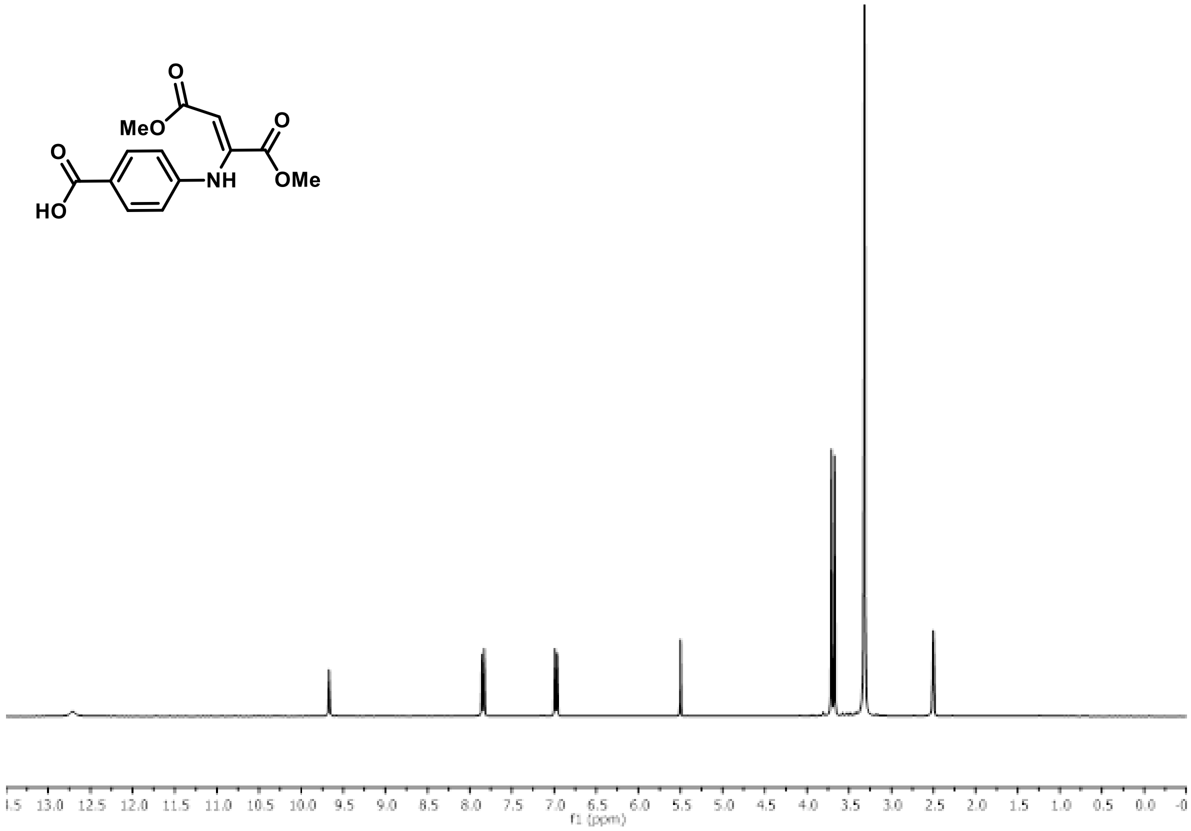

^{1}H NMR spectrum (300 MHz, DMSO-d_6) of **6**.

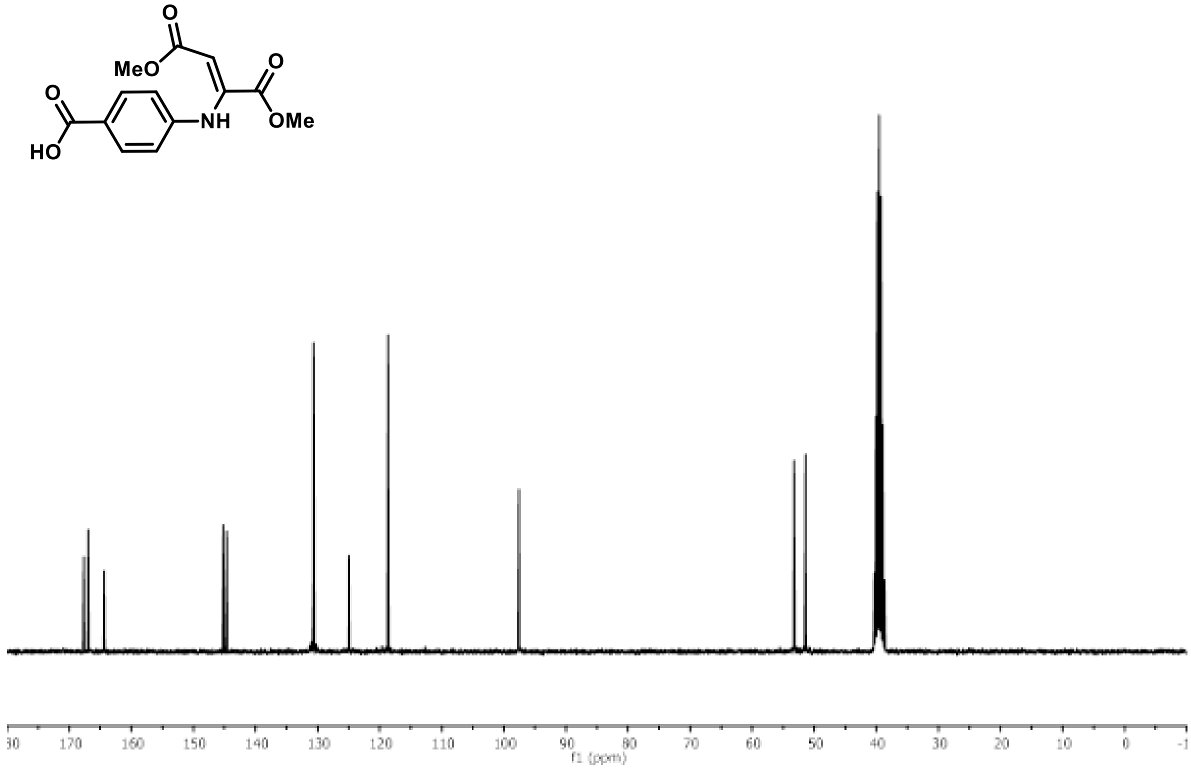

^{13}C NMR spectrum (75 MHz, DMSO-d_6) of **6**.

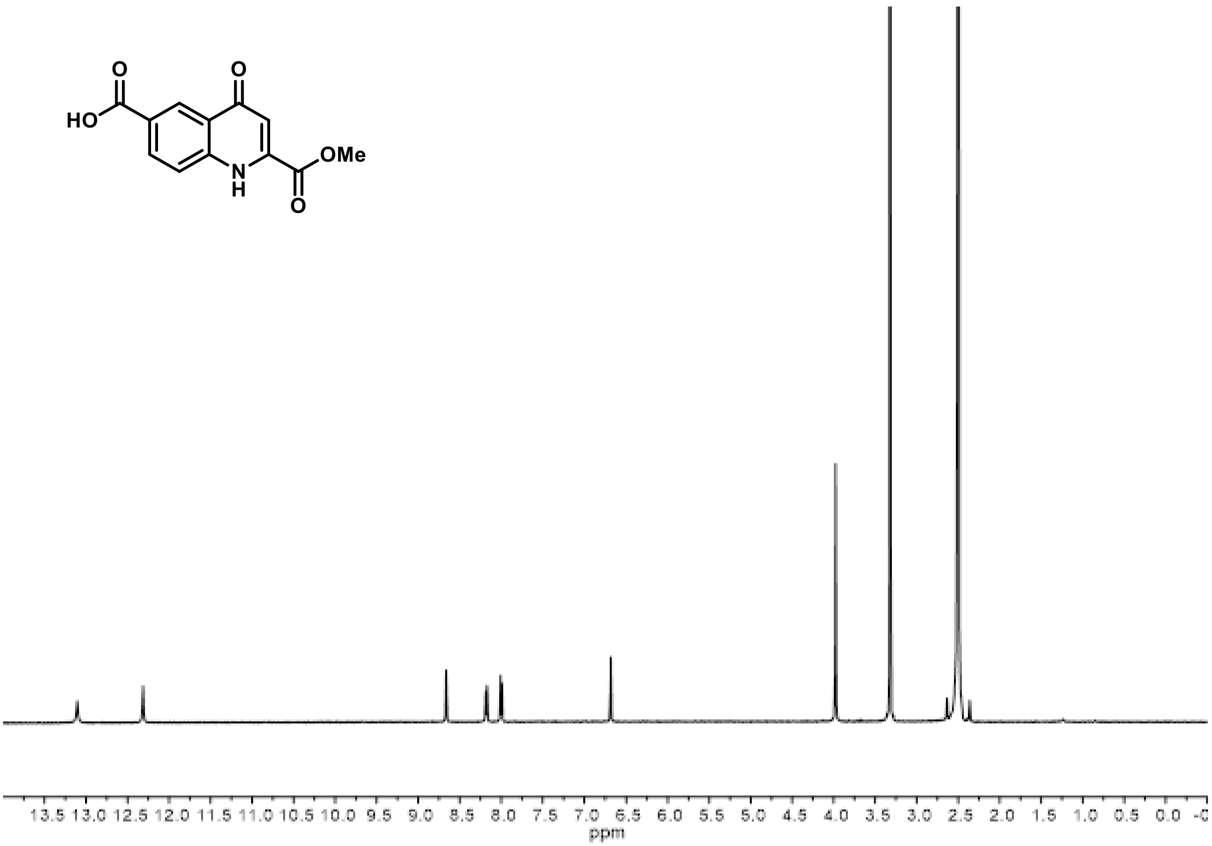

^{1}H NMR spectrum (500 MHz, DMSO-d_6) of **7**.

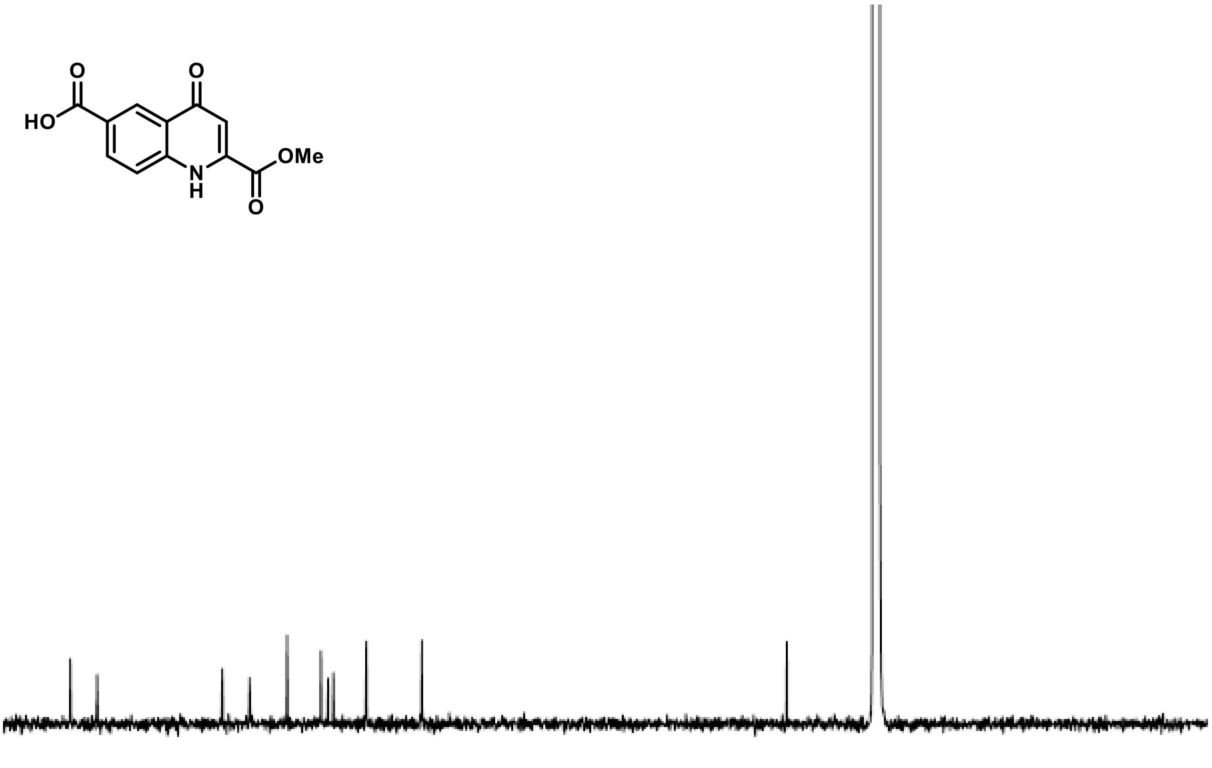

^{13}C NMR spectrum (126 MHz, DMSO-d_6) of **7**.

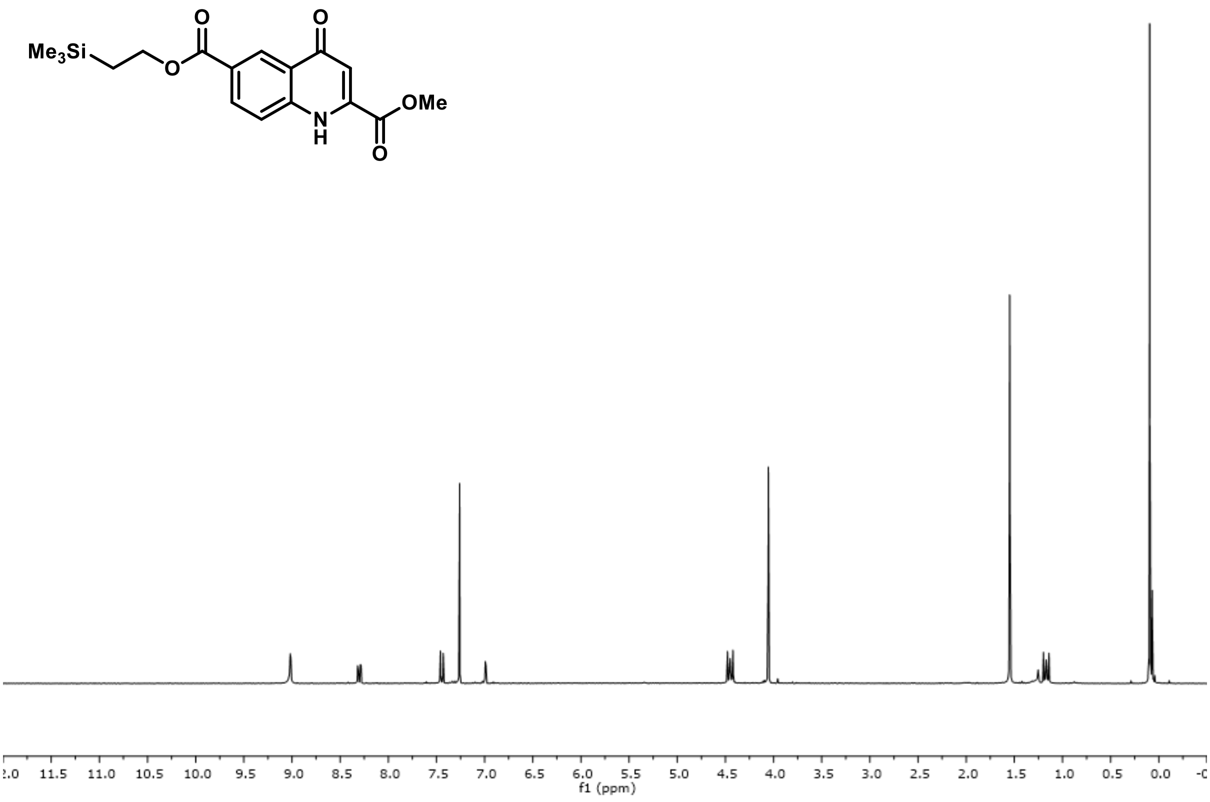

^{1}H NMR spectrum (300 MHz, $CDCl_3$) of **8**.

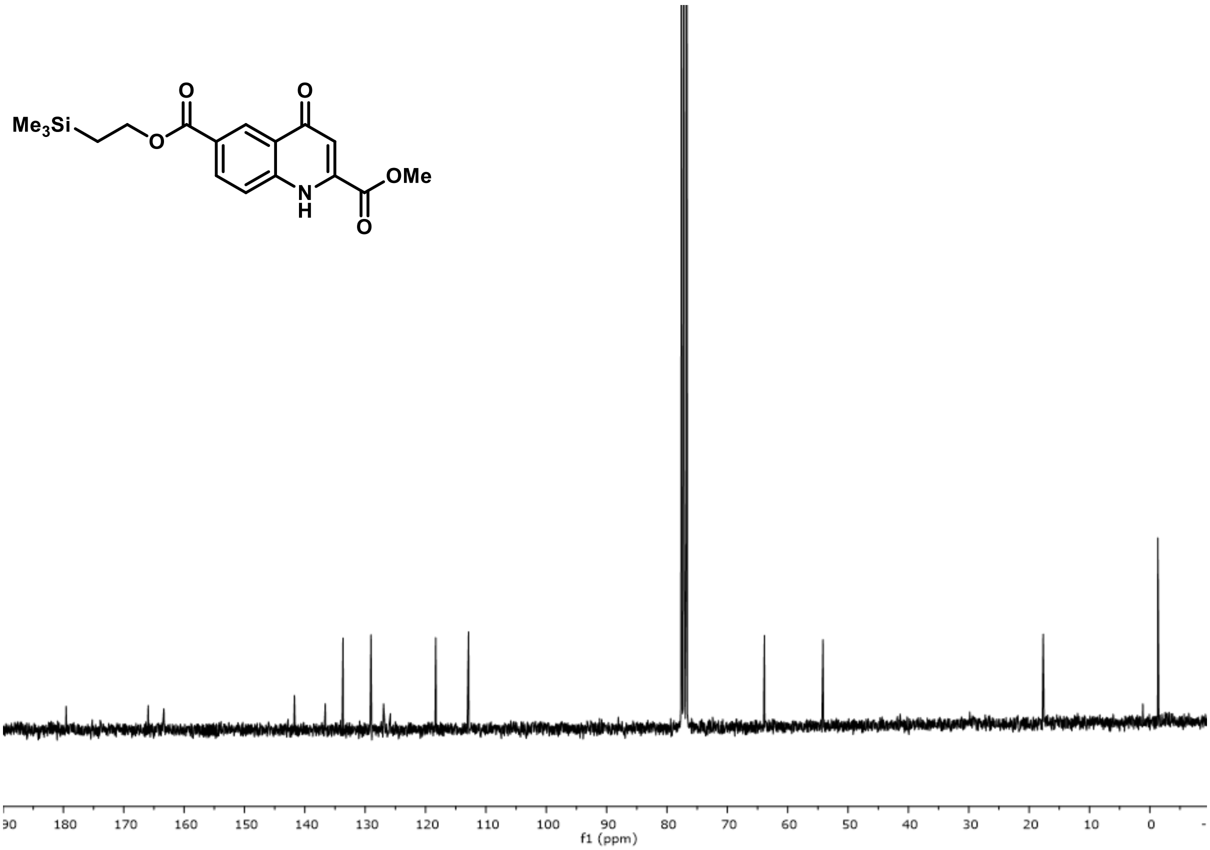

^{13}C NMR spectrum (75 MHz, $CDCl_3$) of **8**.

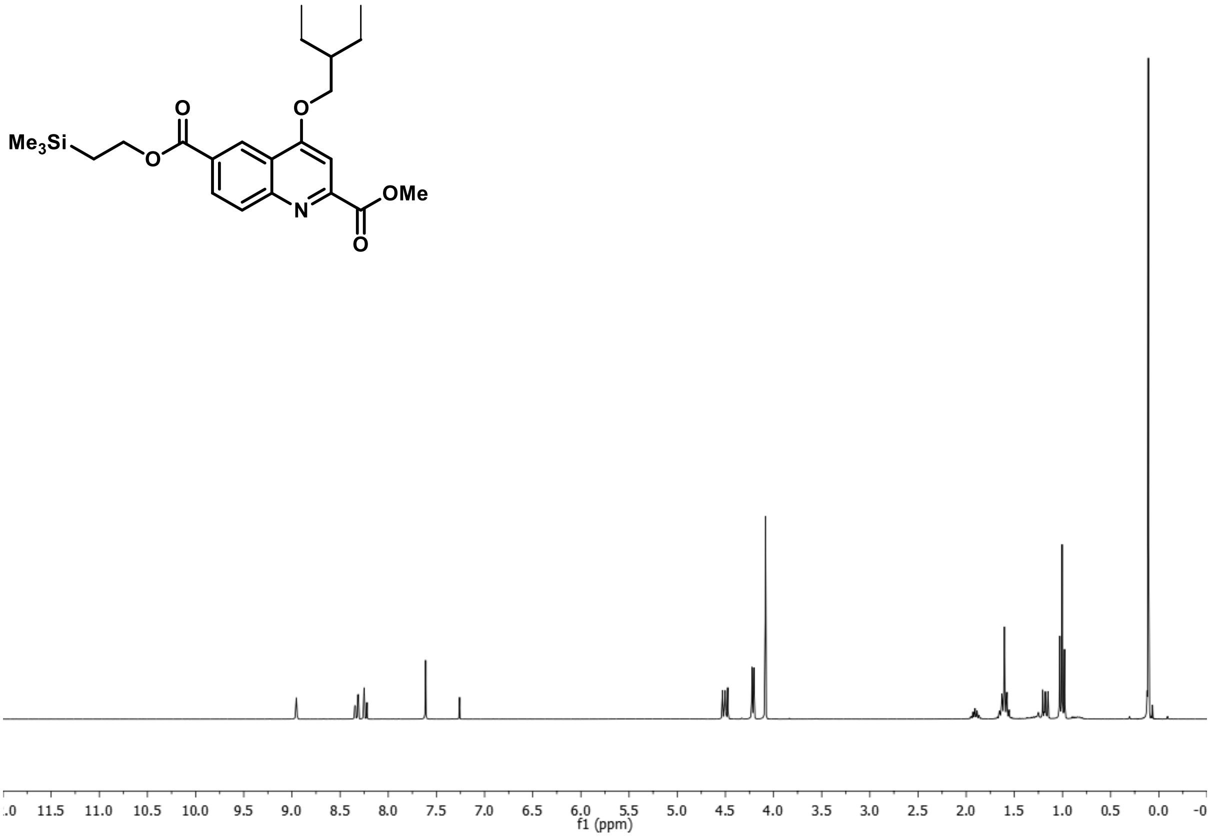

^{1}H NMR spectrum (300 MHz, $CDCl_3$) of **9**.

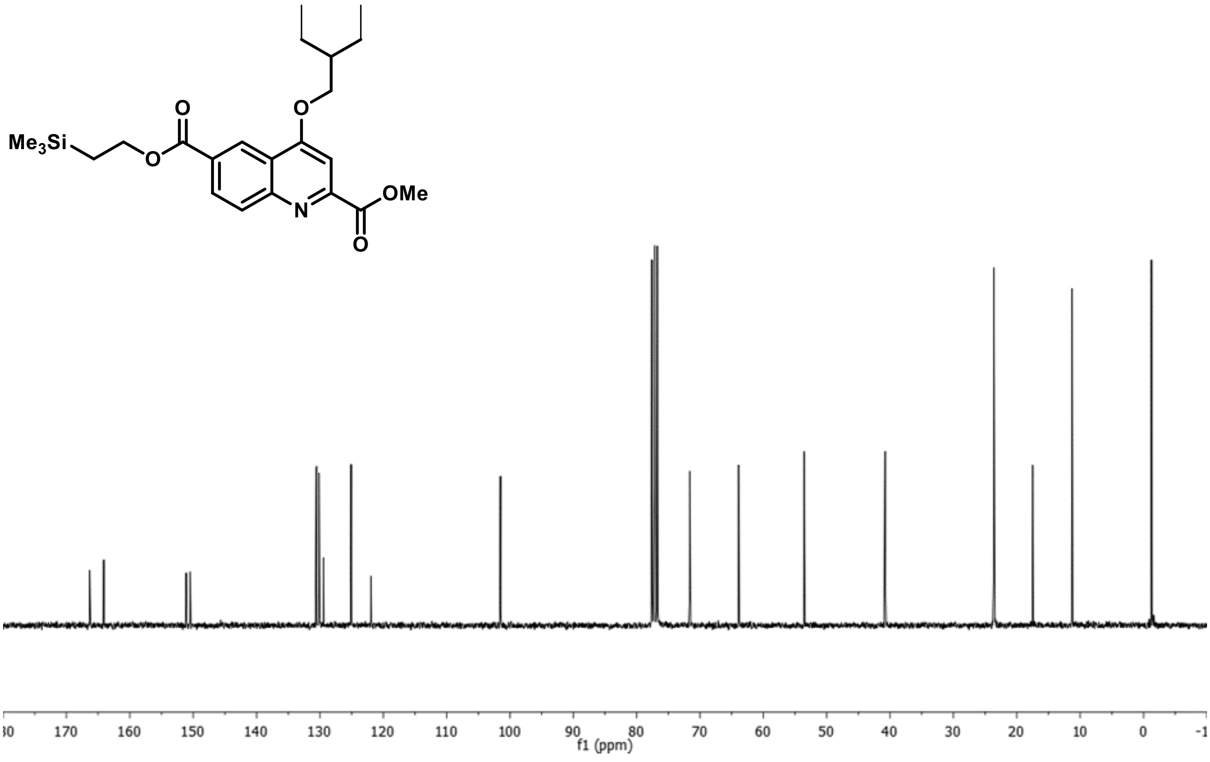

^{13}C NMR spectrum (75 MHz, $CDCl_3$) of **9**.

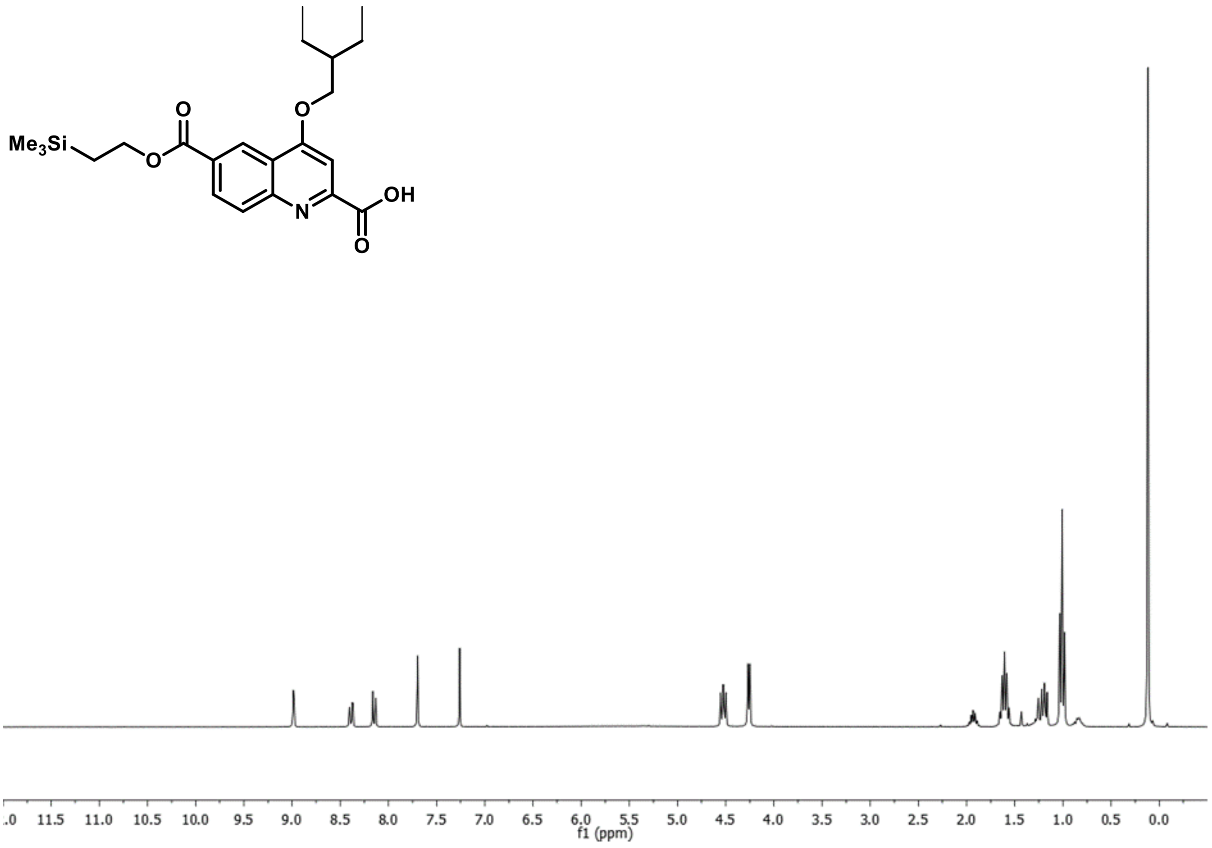

^{1}H NMR spectrum (300 MHz, $CDCl_3$) of **10**.

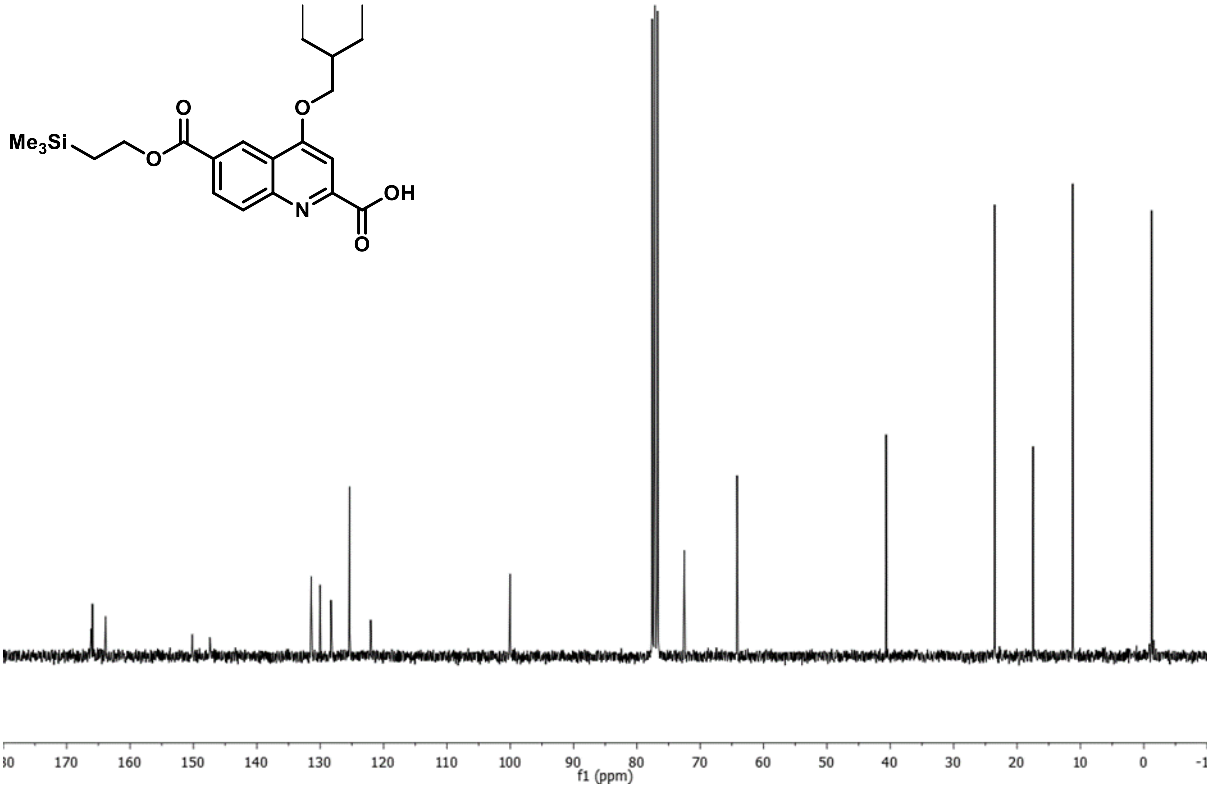

^{13}C NMR spectrum (75 MHz, $CDCl_3$) of **10**.

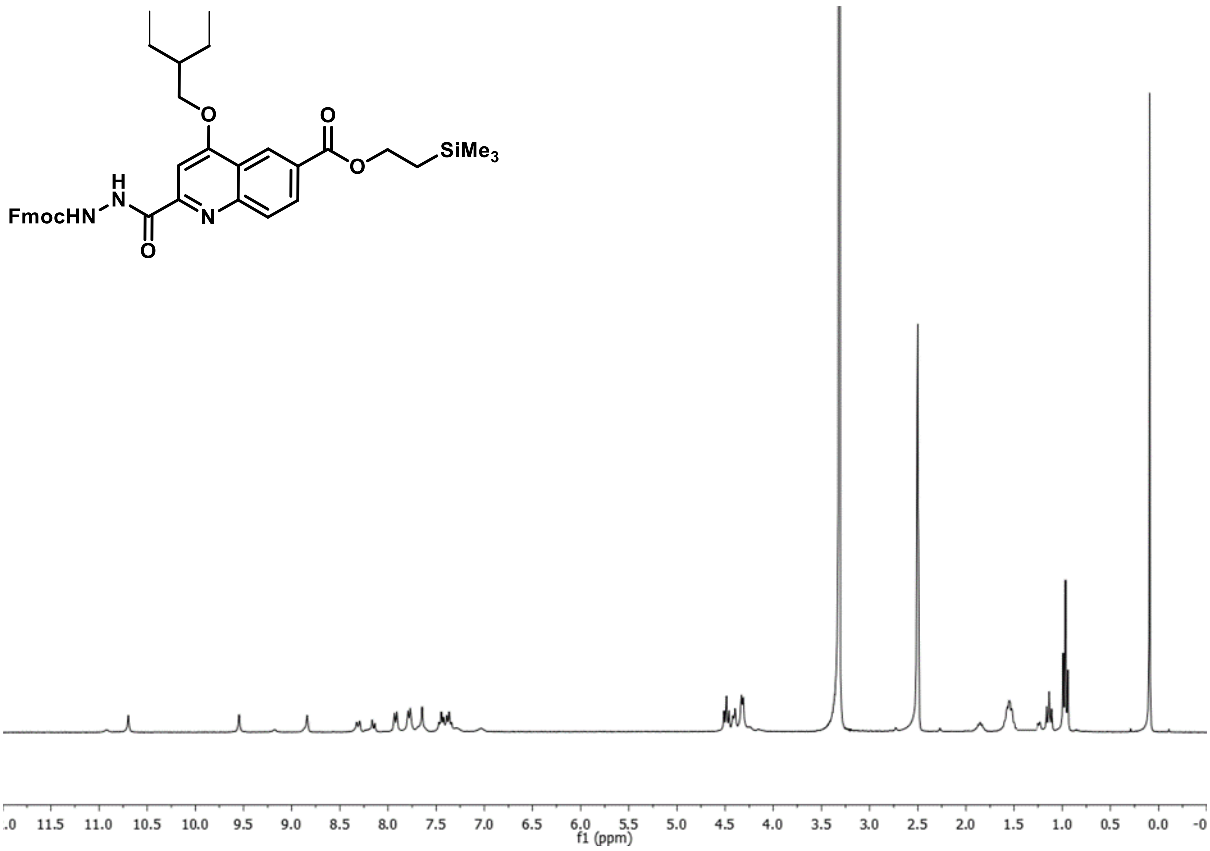

^{1}H NMR spectrum (300 MHz, DMSO-d_6) of **11**.

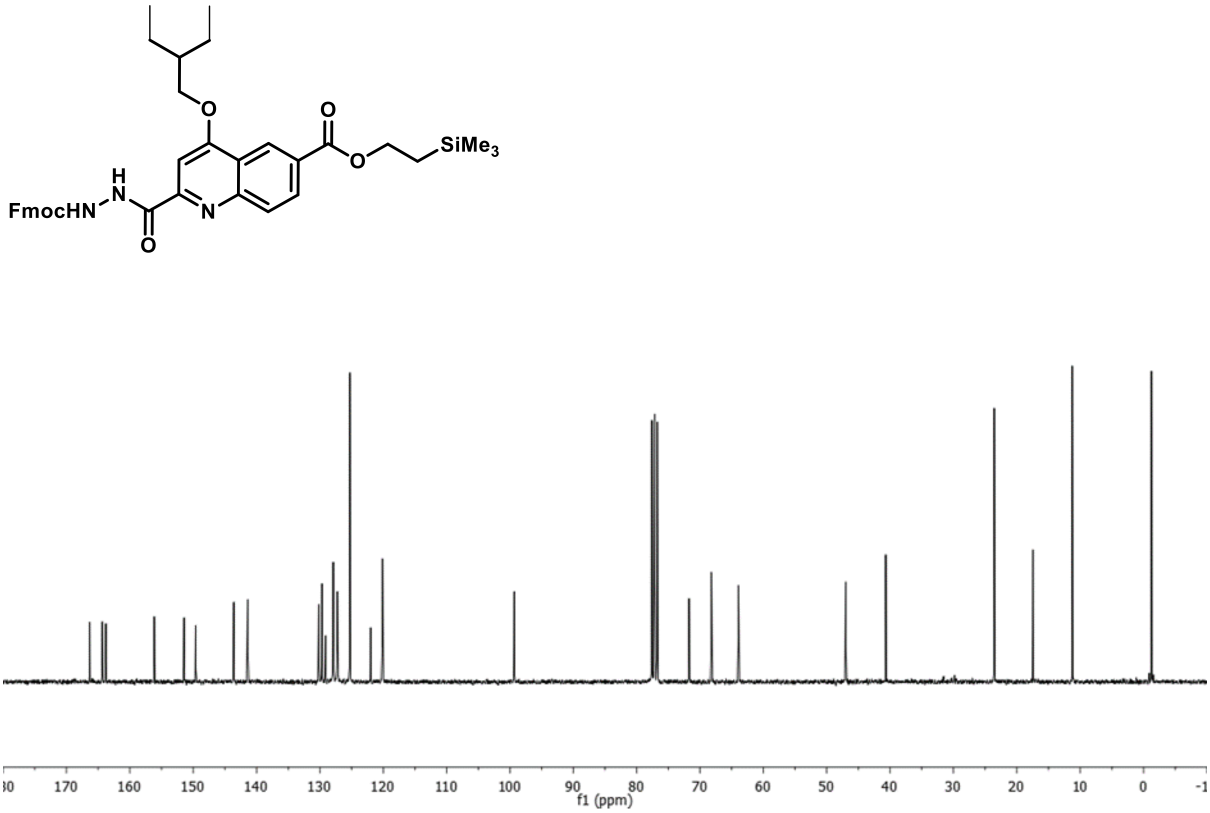

^{13}C NMR spectrum (75 MHz, $CDCl_3$) of **11**.

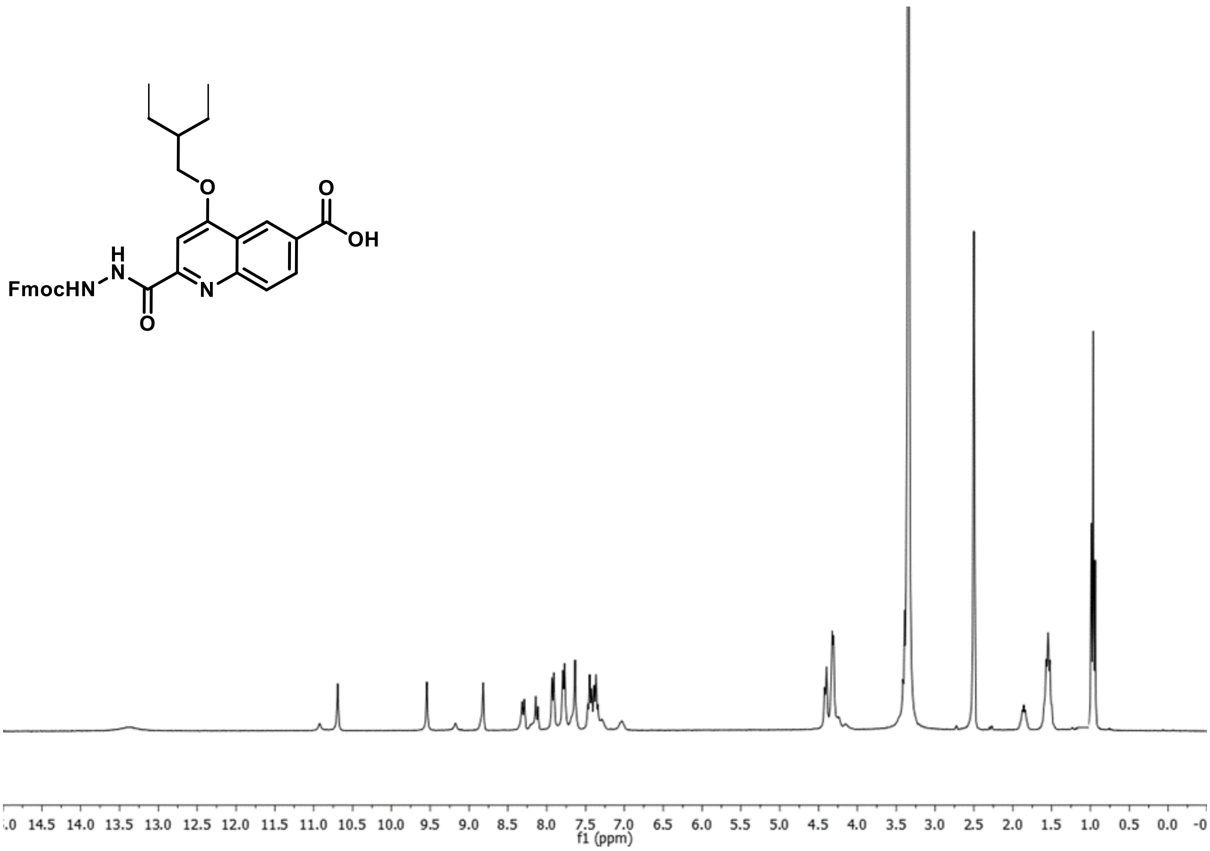

^{1}H NMR spectrum (300 MHz, DMSO-d_6) of **12**.

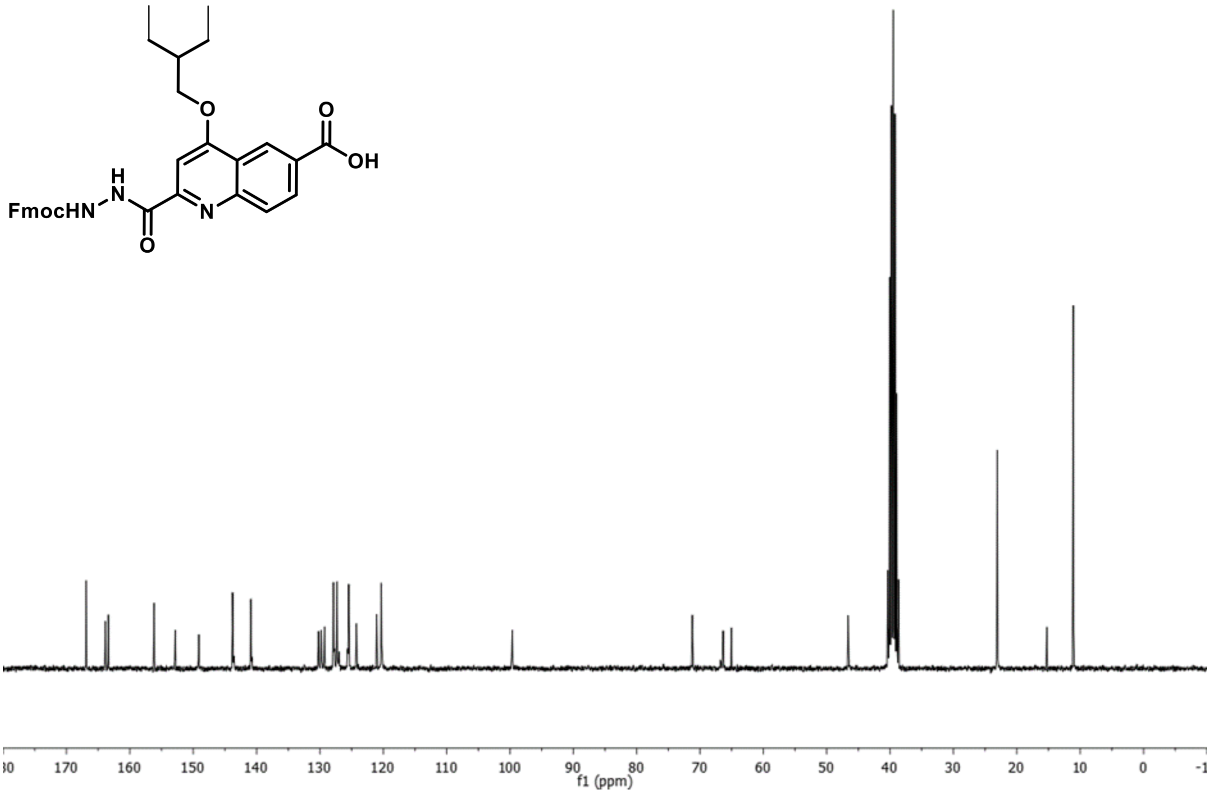

^{13}C NMR spectrum (75 MHz, DMSO-d_6) of **12**.

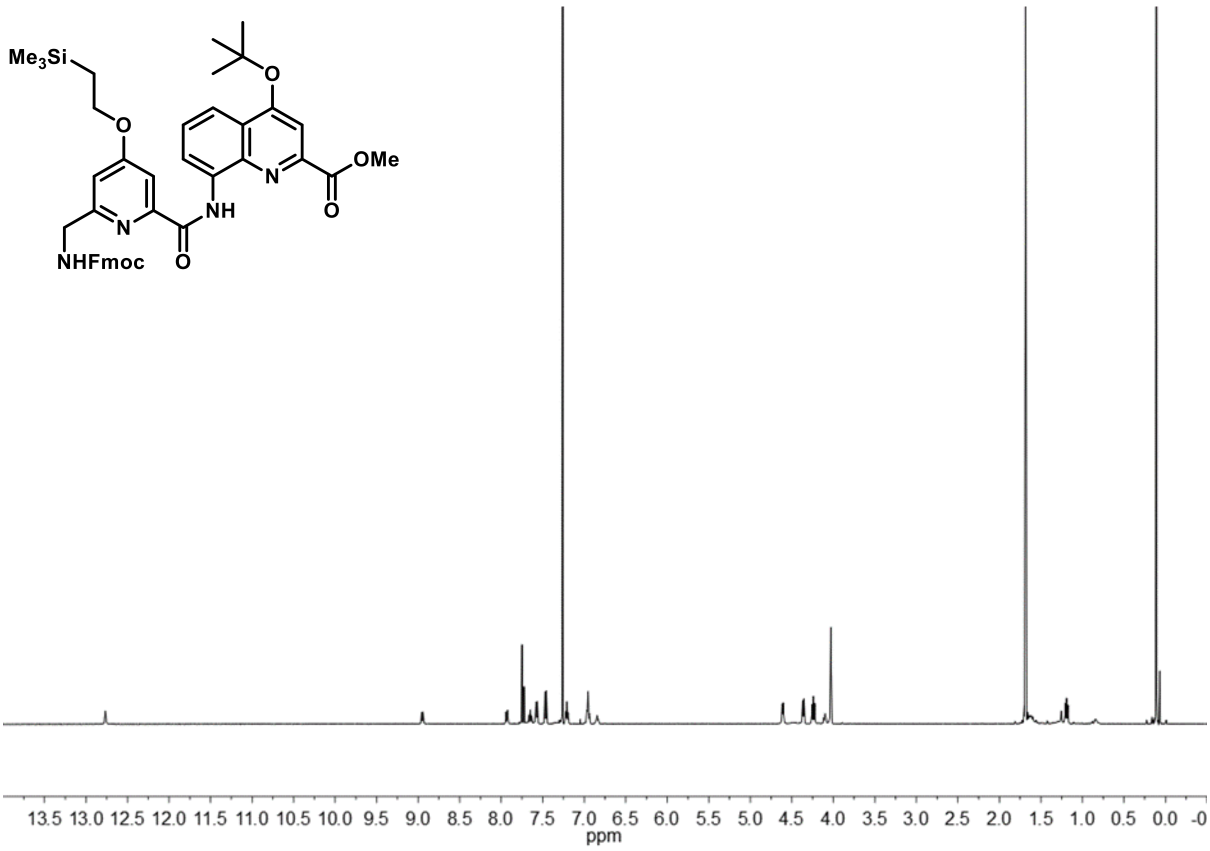

^{1}H NMR spectrum (500 MHz, $CDCl_3$) of **15**.

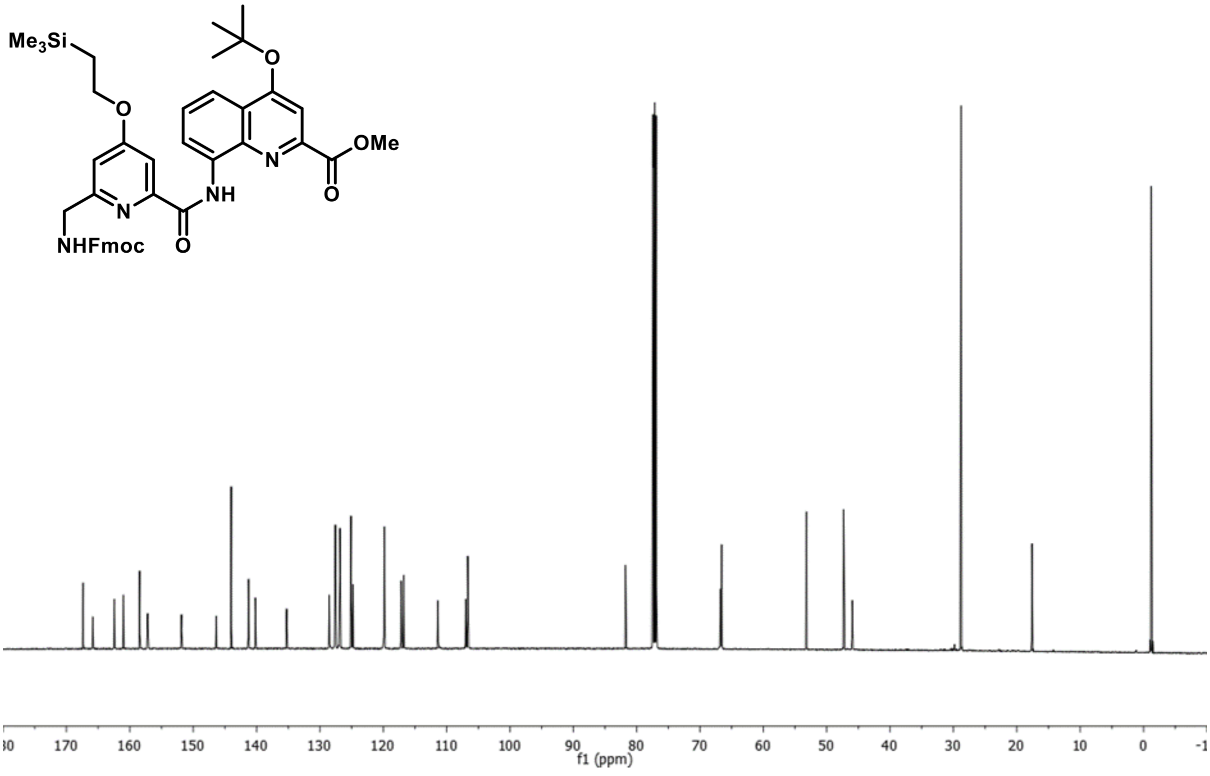

^{13}C NMR spectrum (126 MHz, $CDCl_3$) of **15**.

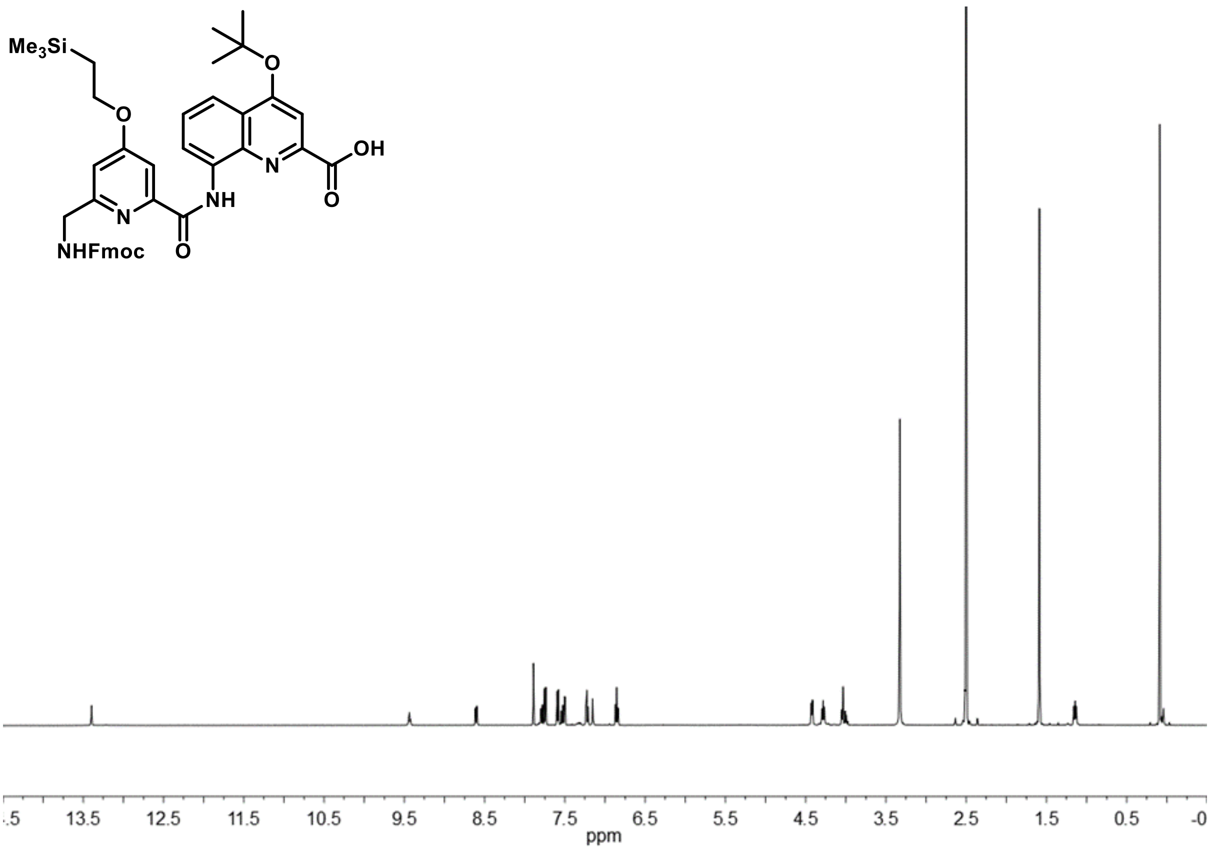

^{1}H NMR spectrum (500 MHz, DMSO-*d*$_6$) of **16**.

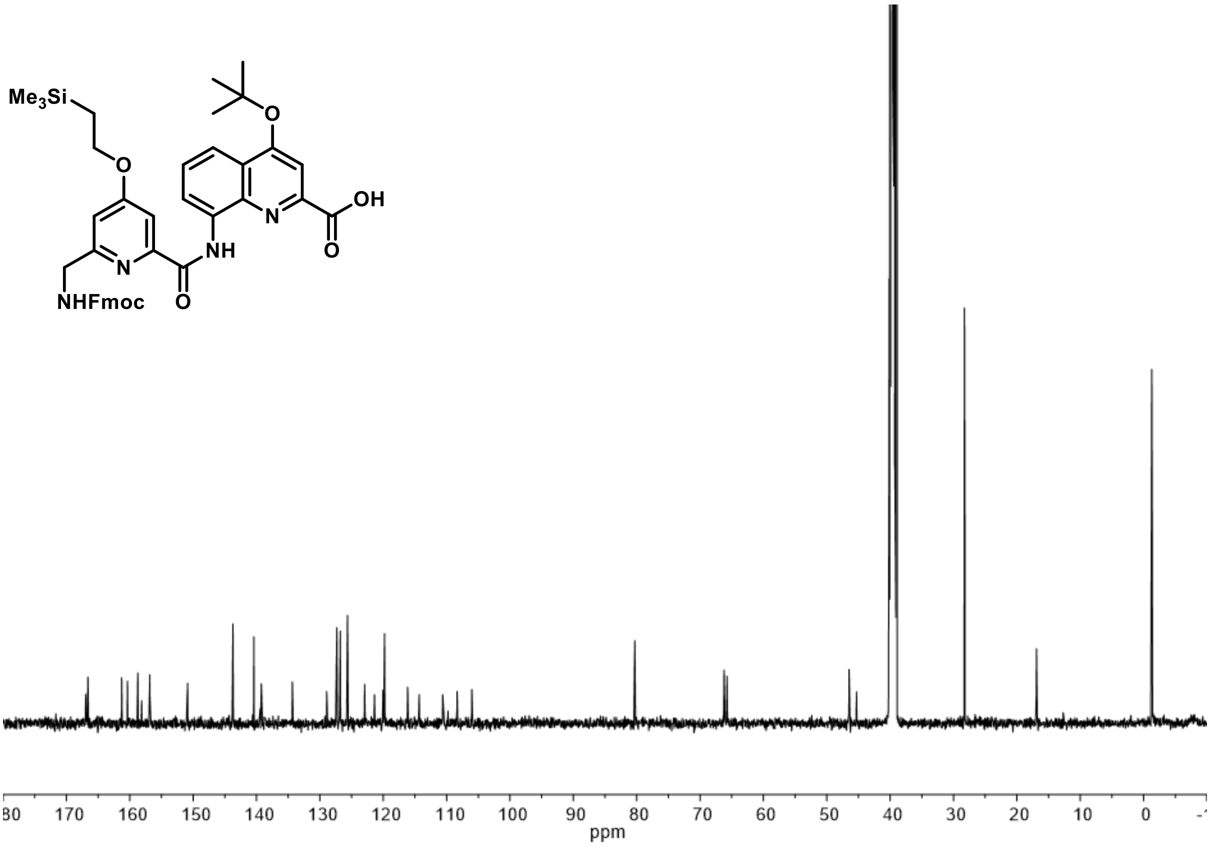

^{13}C NMR spectrum (126 MHz, DMSO-*d*$_6$) of **16**.

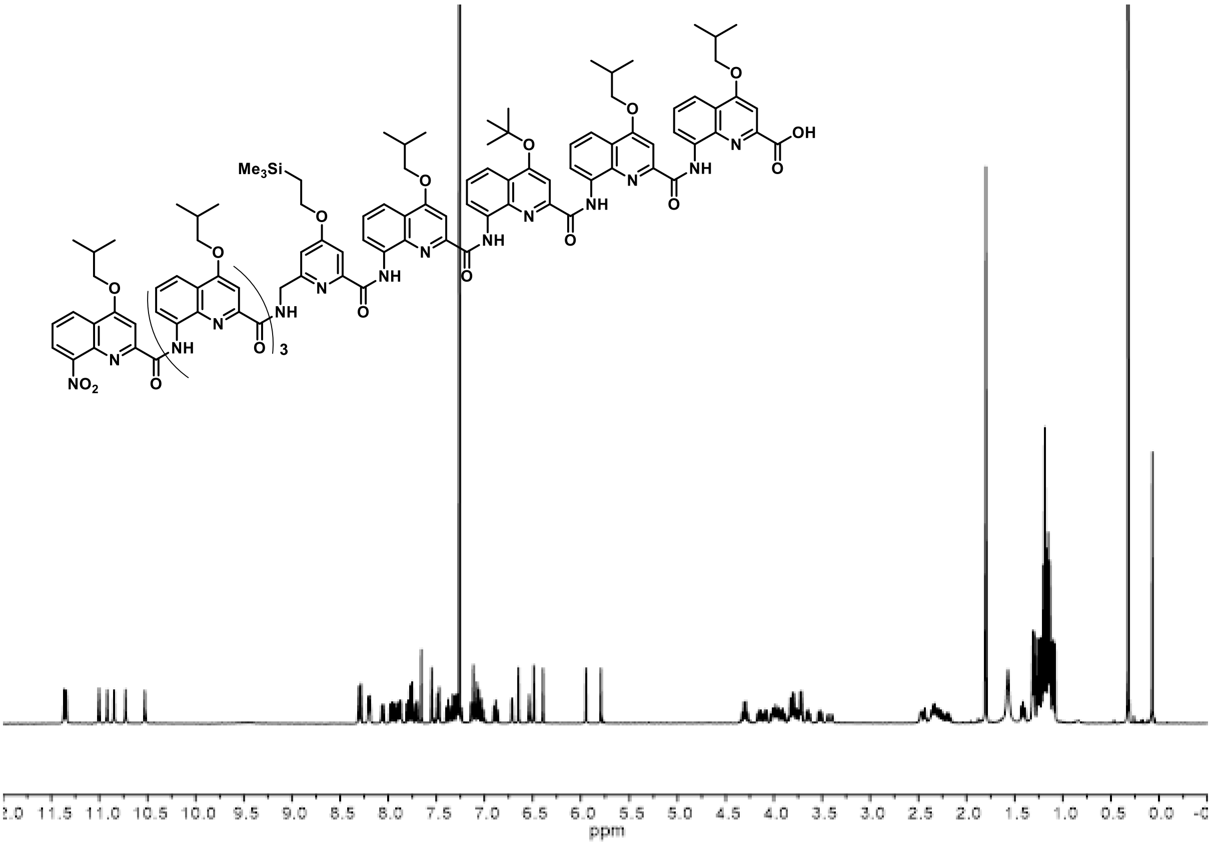

^{1}H NMR spectrum (500 MHz, $CDCl_3$) of **19**.

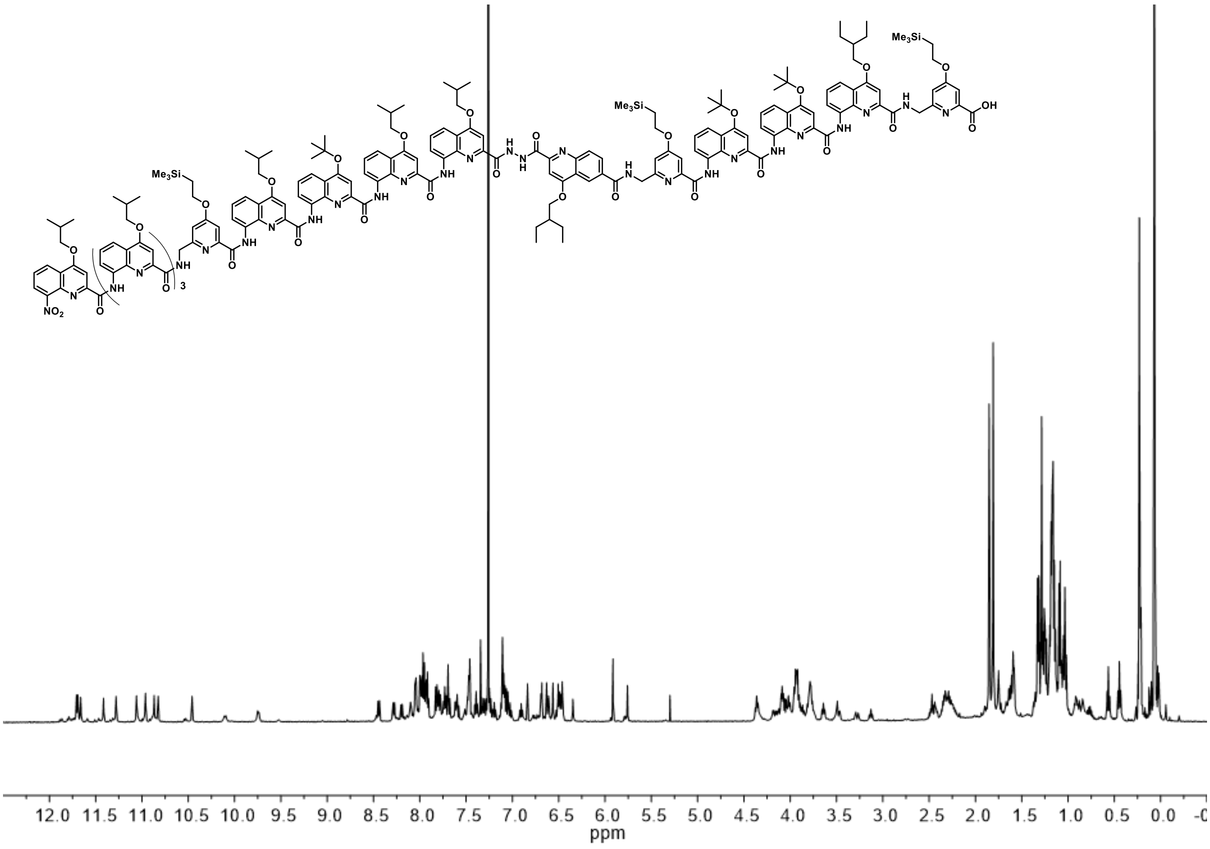

^{1}H NMR spectrum (500 MHz, $CDCl_3$) of **5**.

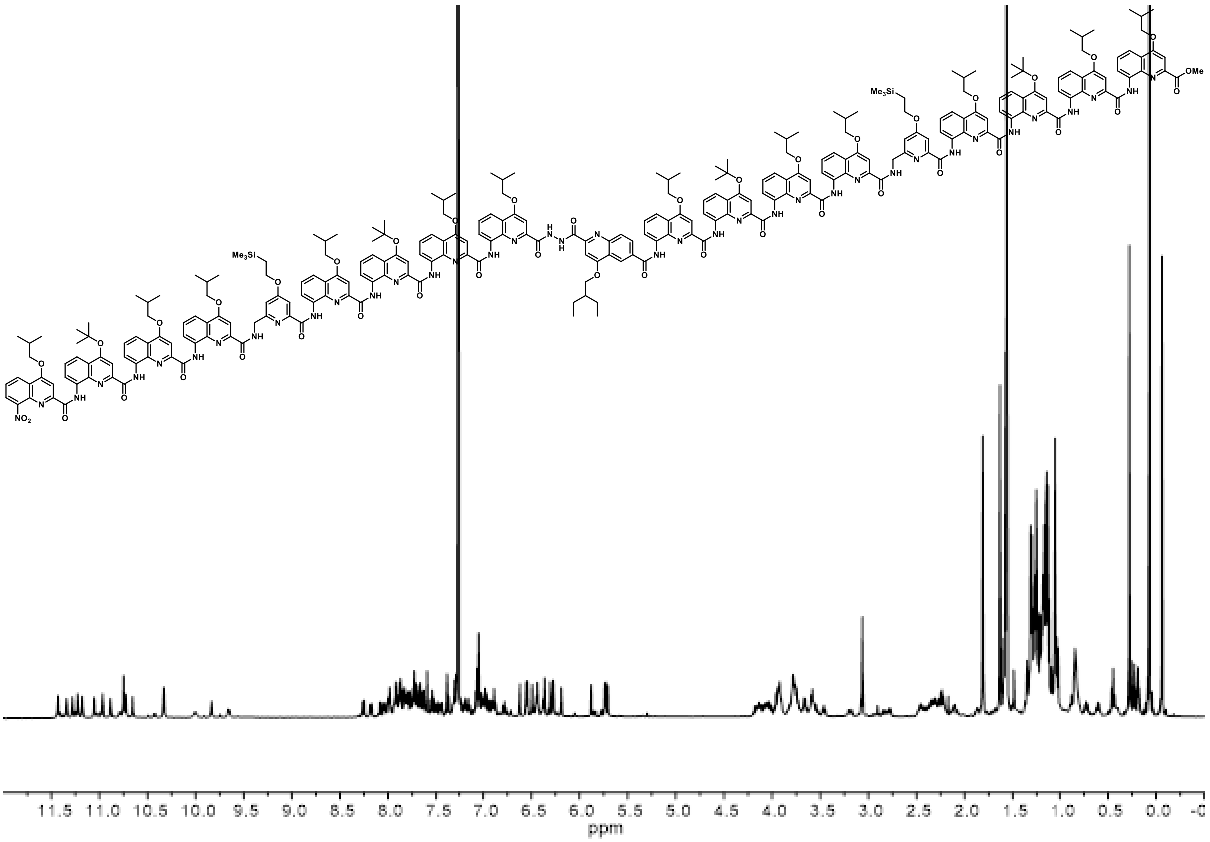

^{1}H NMR spectrum (500 MHz, $CDCl_3$) of **2a**.

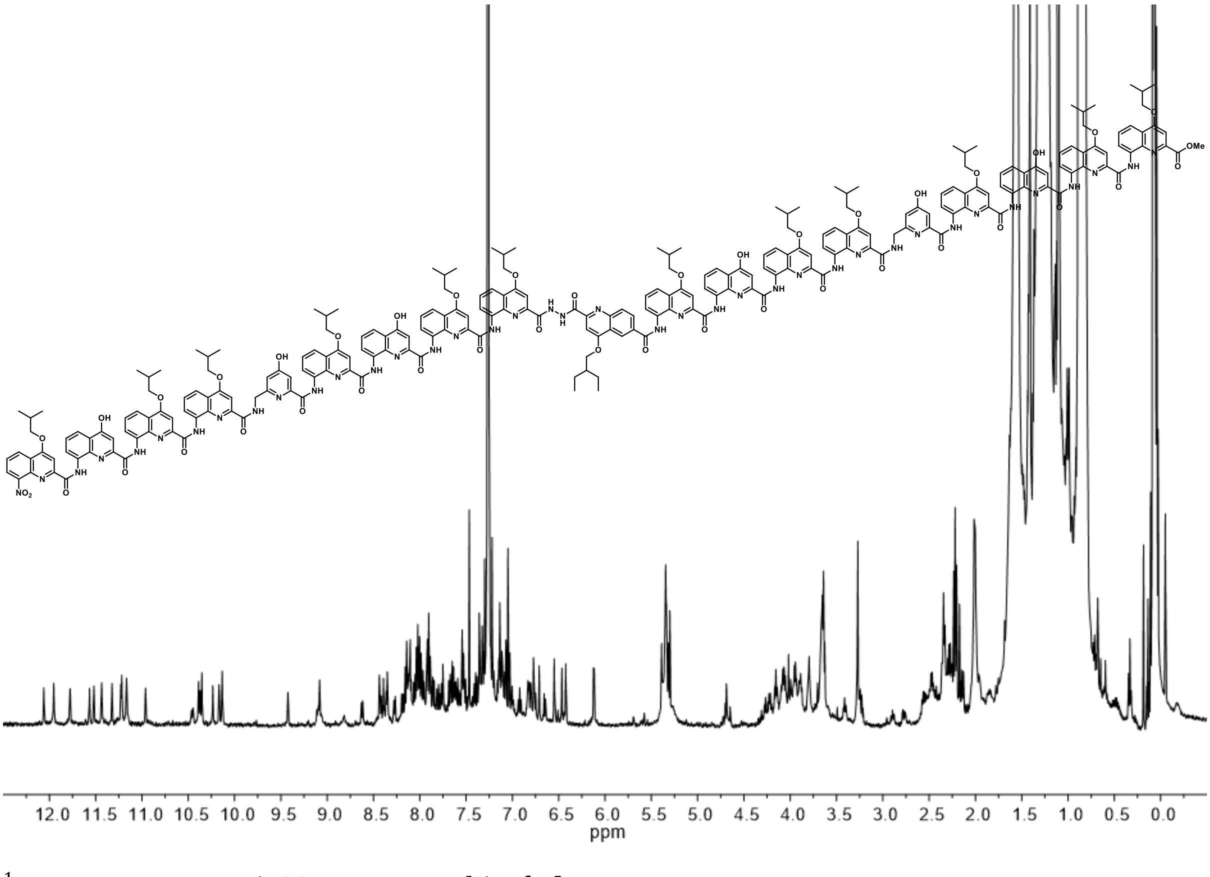

^{1}H NMR spectrum (500 MHz, $CDCl_3$) of **2b**.

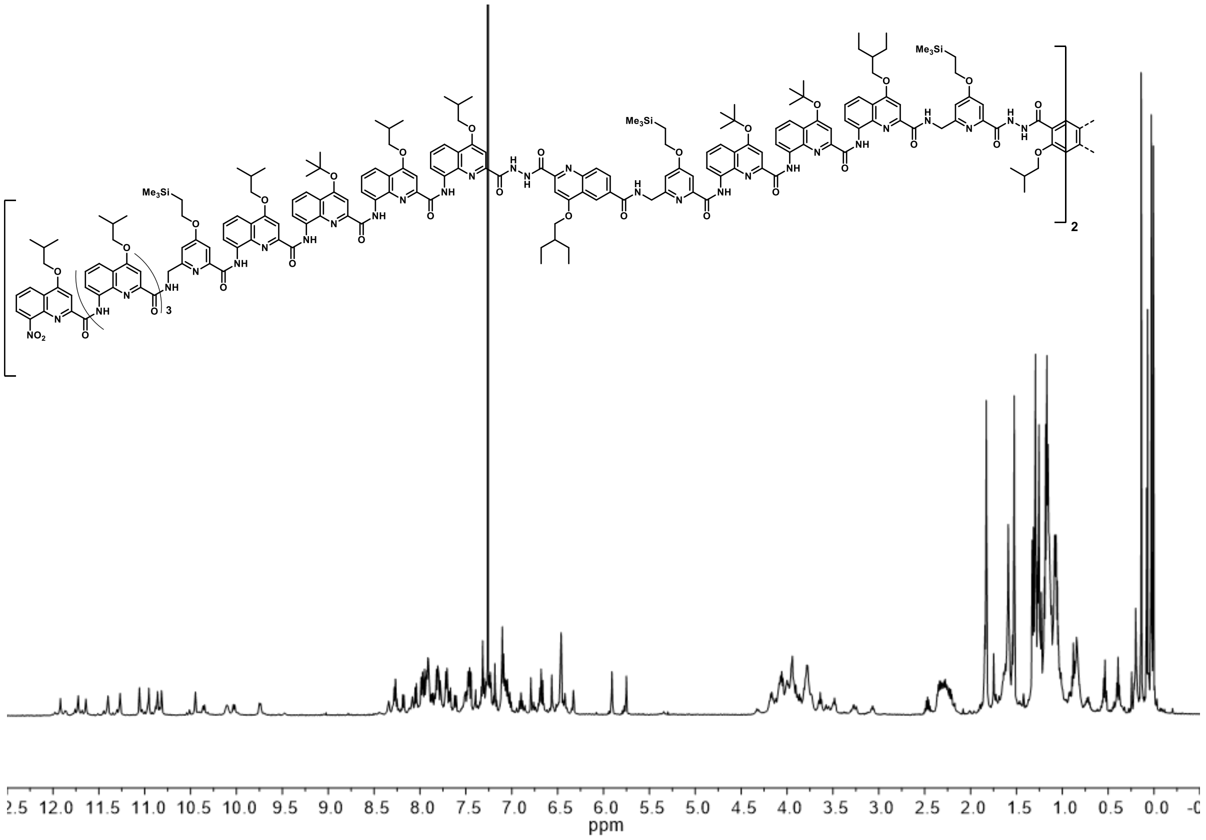

^{1}H NMR spectrum (500 MHz, $CDCl_3$) of **3a**.

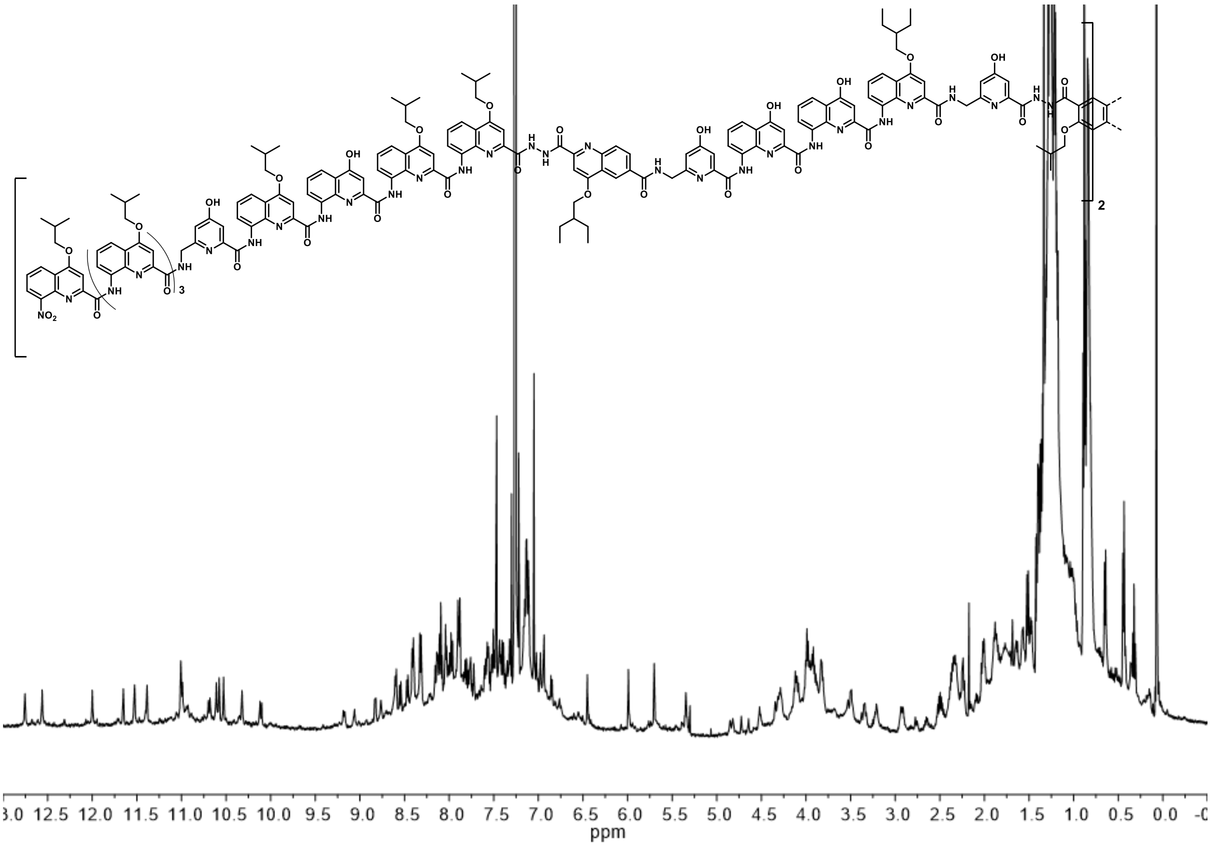

^{1}H NMR spectrum (500 MHz, $CDCl_3$) of **3b**.

7. RP-HPLC analysis of oligomers

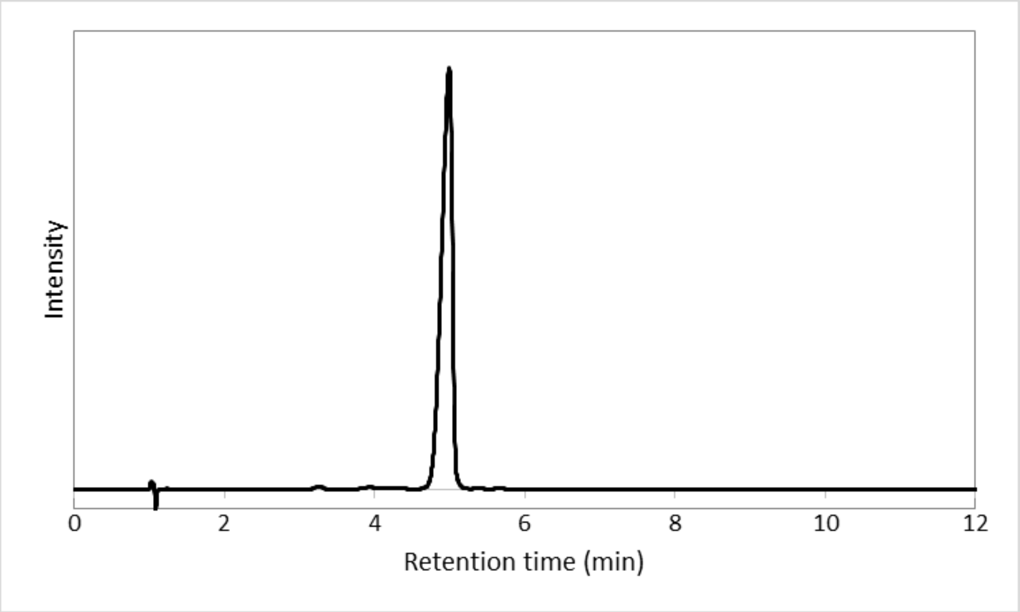

RP-HPLC profile of compound **19**.
